# Anticandidal activity of L-β-(isoxazolin-5-on-2-yl)-alanine due to the inhibition of *O*-acetyl-L-homoserine sulfhydrylase and cystathionine γ-synthase

**DOI:** 10.1080/14756366.2026.2749883

**Published:** 2026-10-21

**Authors:** Aleksandra Kuplińska, Edyta Składanek, Hira Zahoor Rajput, Joanna Stefaniak-Skorupa, Kamila Rząd, Katarzyna Kozłowska-Tylingo, Marek Wojciechowski, Milewska Maria J, Iwona Gabriel, Sławomir Milewski

**Affiliations:** ^a^ Department of Pharmaceutical Technology and Biochemistry, Gdańsk University of Technology, Gdańsk, Poland; ^b^ Department of Organic Chemistry, Gdańsk University of Technology, Gdańsk, Poland

**Keywords:** Methionine biosynthetic pathway, *O*-acetyl-L-homoserine sulfhydrylase, cystathionine γ-synthase, dead-end inhibition, antifungal action

## Abstract

The L-β-(isoxazolin-5-on-2-yl)-alanine (BIA) exhibits *in vitro* growth inhibitory activity against human pathogenic yeasts of Candida spp, with MIC_90_ values in 0.5 – 8 µg/mL range. BIA is transported in *Candida albicans* by the Mup1p permease and then inhibits enzymes of the methionine biosynthetic pathway, *O*-acetyl-L-homoserine sulfhydrylase Met15p and cystathionine γ-synthase Str2p. The slight, borderline synergistic effect was observed for combination of BIA with L-penicillamine. BIA was found to be a competitive inhibitor of Met15p and Str2p, with K_i_ values equal to 0.711 mM and 0.912 mM, respectively. Results of the molecular docking analysis of BIA binding at Met15p active centre suggest that this compound could possibly act as a dead-end inhibitor. The same mechanism of Str2p inhibition by BIA is suggested. BIA is the first identified inhibitor of both Met15p and Str2p, exhibiting moderate growth inhibitory activity against human pathogenic yeasts of the Candida genus.

## GRAPHICAL ABSTRACT

**Figure:**
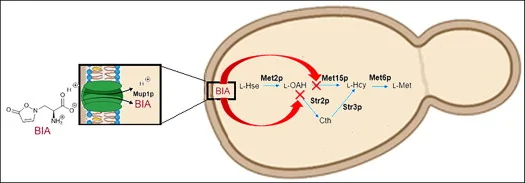

## Introduction

Systemic mycoses, caused mainly in the immunocompromised patients by Candida spp. yeasts, especially *C. albicans,* are difficult to treat because of the small set of effective chemotherapeutics, emerging fungal resistance to some of them, and high mammalian toxicity of another ones^1^^,^^2^. One of the reasons for this situation is a limited number of identified molecular targets that ensure the required selective toxicity of antifungal drugs targeting them. The fungal biosynthetic pathways of nine amino acids (aa) essential for humans, including five constituting the so called aspartate family (L-Asp, L-Asn, L-Met, L-Ile and L-Thr), seem to be an attractive perspective in this respect^3^^,^^4^. One of the human essential aa is L-methionine, formed from L-homoserine or L-serine in the fungal methionine biosynthetic pathway (MBP, Scheme. 1), including the possibility of direct sulfhydrylation with inorganic sulphide or transulfuration with L-cysteine, followed by the subsequent cleavage of cystathionine^5^.

**Scheme 1.**
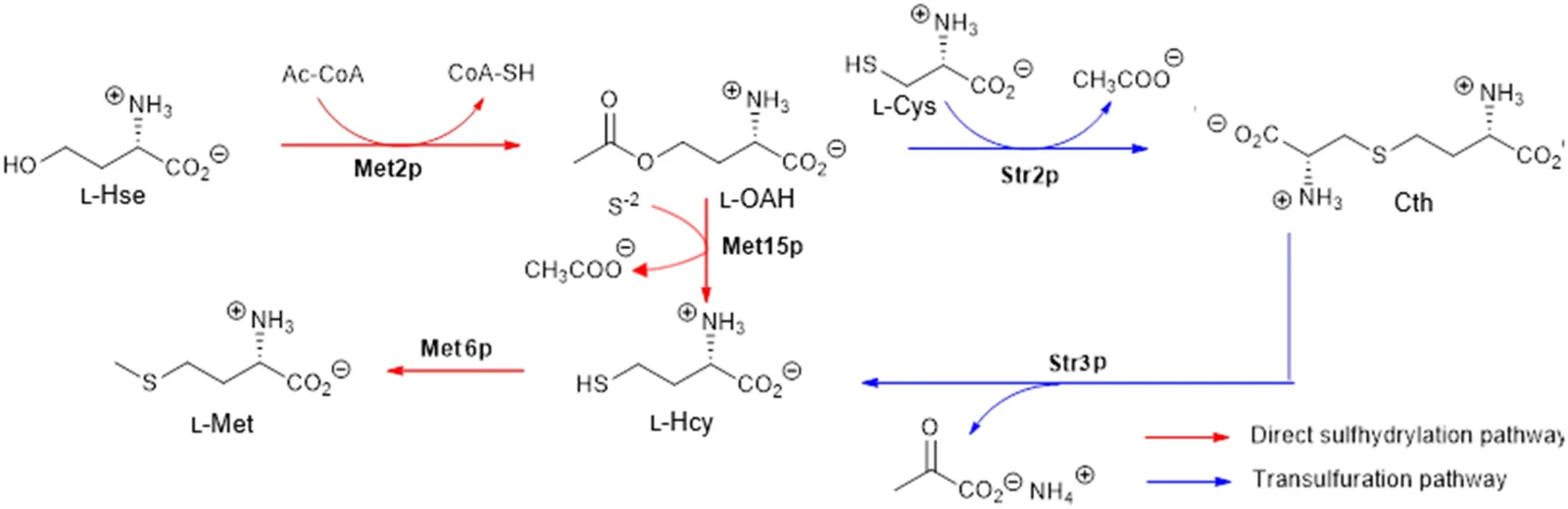
MBP in fungal cells. Met2p, L-homoserine *O*-acetyltransferase; Met15p, *O*-acetyl-L-homoserine sulfhydrylase; Str2p, cystathionine-γ-synthase; Str3p, cystathionine-β-lyase; Met6p, methionine synthase; L-Hse, L-homoserine; L-OAH, *O*-acetyl-L-homoserine; L-Hcy, L-homocysteine; Cth, cystathionine

In most fungal species, the MBP starts with *O*-acetylation of L-homoserine (L-Hse) by L-homoserine *O*-acetyltransferase Met2p, EC 2.3.1.31, affording *O*-acetyl-L-homoserine (L-OAH), followed by a direct sulfhydrylation catalysed by the *O*-acetyl-L-homoserine sulfhydrylase Met15p, EC 4.2.99.10, resulting in L-homocysteine (L-Hcy) formation. However, in some species the latter reaction is performed by the bifunctional *O*-acetyl-L-homoserine/*O*-acetyl-L-serine sulfhydrylase Met17p^6^. Alternatively, L-Hcy can be formed upon transulfuration of L-OAH with L-Cys, affording cystathionine (Cth), catalysed by cystathionine γ-synthase Str2p, EC 2.5.1.48 and subsequent Cth breaking down to pyruvate, ammonium and L-Hcy by cystathionine γ-lyase Str3p, EC 4.4.1.1. L-Hcy is finally converted into L-Met upon methylation by methionine synthase Met6p, EC 2.1.1.14. Two of the enzymes catalysing particular steps of MBP, namely Met2p and Met6p, were proposed as potential antifungal targets^7^^,^^8^. A few inhibitors of the former, demonstrating antifungal activity, were described^9^^,^^10^.

L-β-(Isoxazolin-5-on-2-yl)-alanine (BIA, Figure 1) is a secondary metabolite produced during the seedling stage of the grass pea (*Lathyrus sativus*) and some other legumes^11^. This non-proteinogenic amino acid is the biosynthetic precursor of the neurotoxin β-*N*-oxalyl-L-α,diaminopropionic acid found in *L. sativus*, known to be a cause of human lathyrism^12^. BIA was shown to exhibit growth inhibitory effect on some eukaryotic microorganisms, including baker’s yeast, phytopathogenic fungi and unicellular green algae, while it is not active against bacteria^13^^,^^14^. Mechanism of its antimicrobial effect is not known, although reversal of BIA action on *S. cerevisiae* by L-Met, L-Cys, and L-Hcy^14^ suggests targeting one of the enzymes of MBP.

**Figure 1.**
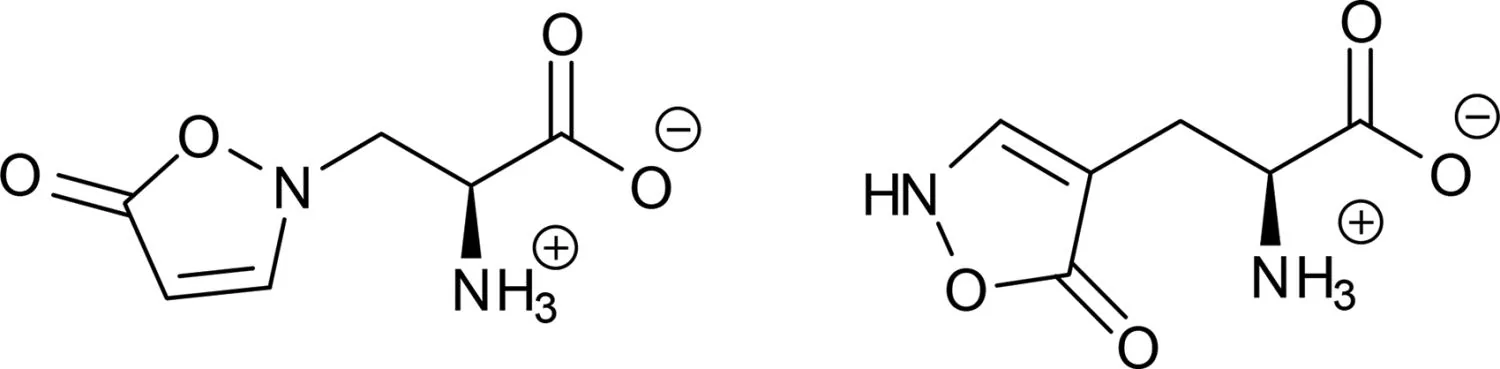
Structures of BIA (left) and its constitutive isomer TAN-950 A (right).

In this work we present results of our studies on growth inhibitory activity of BIA against human pathogenic yeasts of Candida spp. and identification of its molecular target in *Candida albicans.*

## Materials and methods

### Chemistry

#### General aspects

All solvents and reagents were used as purchased from commercial sources.^1^H NMR and ^13^C NMR spectra were obtained at 500 MHz Varian Unity Plus spectrometer and the deuterated solvents were used as internal locks. HRMS spectra were recorded with Agilent 6540 UHD QTOF-MS system. Column chromatography was performed with silica gel (0.040–0.063 mm) by using the indicated solvent systems. Automated flash chromatography was performed on CombiFlash Nextgen 300+ (Teledyne ISCO) system using RediSep Bronze silica gel column cartridges.

#### Syntheses

*1-O-(tert-Butoxycarbamoyl)-N-(tert-butoxycarbonyl)-β-chloro-L-alanine* **1**. 1-Ethyl-3-[3-(dimethylamino)propyl]carbodiimide hydrochloride (826 mg, 4.3 mmol) was added to a stirred solution of *N*-(*tert*-butoxycarbonyl)-β-chloro-L-alanine (1 g, 4.3 mmol) and *N*-(*tert*-butoxycarbonyl)-hydroxylamine, prepared by the method of Harris^15^, (572 mg, 4.3 mmol) in anhydrous dichloromethane (50 ml) and the solution was stirred for 15 h. The solvent was evaporated *in vacuo* and the resulting oil dissolved in ethyl acetate (50 ml), washed with water (2 × 30 ml) and dried (Na_2_SO_4_). The solvent was evaporated *in vacuo* to give **1** as a white solid (1.25 g, 86%).^1^H NMR (500 MHz, CDCl_3_): δ = 1.45 (s, 9H, (C*H*_3_)_3_C-), 1.49 (s, 9H, (C*H*_3_)_3_C-), 3.88 (dd, 1H, J 11.0, 4.0 C*H*_2_-CH), 4.0 (dd, 1H, J 11.0, 4.0 C*H*_2_-CH), 4.87 (m, 1H, CH_2_-C*H*), 5.41 (d, 1H, -N*H*-CO-), 7.96 (s, 1H, -N*H*-O-); ^13^C NMR (500 MHz, CDCl_3_): δ = 27.96 ((*C*H_3_)C), 28.21 ((*C*H_3_)C), 44.75 (*C*H_2_-CH) 53.29 (CH_2_-*C*H), 80.96 ((CH_3_)*C*), 83.87 ((CH_3_)*C*), 154.82 (NH-*C*O), 154.95 (NH-*C*O), 168.93 (CH-*C*O_2_).

*α-(N-tert-Butoxycarbonyl)-β-(N-tert-butoxycarbonyl, N-hydroxy)-β-amino-L-alanine* **2**. Sodium hydride (140 mg of a 60% suspension in mineral oil, 3.5 mmol, 1.0 eq.) was added to a stirred solution of compound **1** (1.2 g, 3.5 mmol) in anhydrous dimethylformamide (50 ml) at 0 °C, and the suspension was stirred for 3 h. A solution of Cs_2_CO_3_ (685 mg, 2.1 mmol, 0.6 eq.) in water (10 ml) was then added, and the solution was stirred for 1 h and then washed with diethyl ether (2 × 10 ml), acidified with KHSO_4_ solution (1 M aq.) and extracted with ethyl acetate (3 × 20 ml). The ethyl acetate fractions were combined, washed with water (3 × 10 ml), dried (Na_2_SO_4_) and the solvent was evaporated *in vacuo,* to give the **2** as a yellow oil, which was used for the next step without purification. ^1^H NMR (500 MHz, CDCl_3_): δ = 1.42 (s, 9H, (C*H*_3_)_3_C-), 1.45 (s, 9H, (C*H*_3_)_3_C-), 3.75 (dd, 1H, J 11.0, 4.0 C*H*_2_-CH), 3.92 (dd, 1H, J 11.0, 4.0 C*H*_2_-CH), 4.59 (m, 1H, CH_2_-C*H*), 5.49 (d, 1H, -N*H*-CO-).

*α-(N-tert-Butoxycarbonyl)-β-(N-tert-butoxycarbonyl, N-hydroxy)-β-amino-L-alanine benzhydryl ester* **3**. A solution of diphenyldiazomethane, prepared by the method of Klimovica et al.^16^, (485 mg, 2.5 mmol, 1.0 eq.) in acetonitrile (30 ml) was added dropwise to a stirred solution of compound **2** (800 mg, 2.5 mmol) in acetonitrile (10 ml) until the purple diphenyldiazomethane solution was no longer decolourised on addition. The solution was stirred for a further 3 h and then decolourised by the addition of one drop of acetic acid. The solvent was removed *in vacuo* and the resulting oil purified by flash chromatography, eluting with 20% ethyl acetate/hexane. The product **3** was obtained as an oil (850 mg, 70%). ^1^H NMR (500 MHz, CDCl_3_): δ = 1.42 (s, 9H, (C*H*_3_)_3_C-), 1.46 (s, 9H, (C*H*_3_)_3_C-), 3.75 (dd, 1H, C*H*_2_-CH), 3.93 (dd, 1H, C*H*_2_-CH), 4.76 (m, 1H, CH_2_-C*H*), 5.40 (d, 1H, -N*H*-CO), 6.93 (s, 1H, C*H*-Ph_2_), 7.23–7.4 (m, 10H, 2xPh), 8.1 (s, 1H, N-O*H*)

*α-(N-tert-Butoxycarbonyl)-β-(N-tert-butoxycarbonyl, N-propioloxy)-β-amino-L-alanine benzhydryl ester* **4**. EDCI hydrochloride, (380 mg, 1.97 mmol, 1.2 eq.) was added to a stirred solution of compound **3** (800 mg, 1.64 mmol) and propiolic acid (184 mg, 2.62 mmol, 1.6 eq.) in anhydrous dichloromethane (15 ml), and the solution was stirred for 18 h, with the formation of a white suspension in a brown solution. The solvent was evaporated *in vacuo* and the residue suspended in ethyl acetate (10 ml). The suspension was filtered, and the filtrate concentrated *in vacuo* to give a brown oil. Purification by flash chromatography, eluting with 30% ethyl acetate/hexane gave **4** as a white solid (740 mg, 84%). ^1^H NMR (500 MHz, DMSO-d): δ = 1.36 (s, 9H, (C*H*_3_)_3_C-), 1.37 (s, 9H, (C*H*_3_)_3_C-), 3.48 (C≡C*H*), 3.84 (m, 1H, C*H*_2_-CH), 4.0 (m, 1H, C*H*_2_-CH), 4.36 (m, 1H, CH_2_-C*H*), 4.95 (d, 1H, -N*H*-CO), 6.74 (s, 1H, C*H*-Ph_2_), 7.21.-7.45 (m, 10H, 2xPh); ^13^C NMR (500 MHz, DMSO-d): δ = 27.95 ((*C*H_3_)C), 28.54 ((*C*H_3_)C), 52.12 (*C*H_2_-CH) 60.12 (CH_2_-*C*H), 72.37 (C≡*C*H), 77.57 (*C*HPh_2_), 79.01 ((CH_3_)*C*), 83.31 ((CH_3_)*C*), 81.4 (*C*≡CH), 125.7, 128. 21, 128.81 (2xPh) 140.49, 140.50 (aromatic *ipso*), 153.82, 153.99 (2 x NH-*C*O), 155.69 (*C*O-C≡CH) 169.2 (CH-*C*O_2_).

*L-β-(Isoxazolin-5-on-2-yl)-alanine (BIA)* **5**. The ester **4** (700 mg, 1.3 mmol) was dissolved in 98% formic acid (10 ml) and solution was stirred at 35 °C for 18 h. The formic acid was removed *in vacuo* and the residue dissolved in water (15 ml) and washed with diethyl ether (2 × 5 ml). The aqueous layer was freeze dried to give a yellow solid, which was purified using column chromatography, eluting with DCM/methanol/water 7:4:1. The final product **5** was obtained as a white solid (80 mg, 36%), m. p. 202 – 204 °C, decomp. (lit. 203 – 205 °C, decomp.); [α]^20^_D_ −59.3° (c = 0.8, H_2_O)/lit. [α]^20^_D_ −62.7° (c = 1.4, H_2_O)/; ^1^H NMR (500 MHz, D_2_O): δ = 3.98 (t, 1H, C*H-*CH_2_), 4.18 (d, 2H, CH-C*H_2_*), 5.15 (d, 1H, J 3.5, C*H*=CH), 8.12 (d, J 3.5, CH = C*H*); ^13^C NMR (125 MHz, D_2_O): δ = 52.85, 53.16, 87.00, 154.04, 171.83, 174.46; HRMS (ESI+): calcd. for C_6_H_9_N_2_O_4_ [M + H]^+^ m/z: 173.0557, found m/z: 173.0559.

#### HPLC-DAD analysis

Purity of the final BIA preparation was determined using the Shimadzu LC Nexera 2 HPLC-DAD system consisted of two pumps, autosampler, column oven, and SPD-M40 diode array detector. Detection was carried out at a wavelength of 254 nm. Separation was performed on a Zorbax XDB-C8 column (150 × 4.6 mm, 5 µm particle size). The column temperature was maintained at 30 °C, the flow rate was set at 1.5 ml/min, and the injection volume was 10 µL. The mobile phase consisted of water containing 0.05% formic acid as component A and acetonitrile as a component B. The chromatographic run was performed in gradient mode, with the proportion of component B increasing from 5% to 65% over 5 min.

### Determination of biological properties

#### Microorganisms

*Escherichia coli* ER2566 (New England Biolabs, MA, USA) cells were used as a host for expression of the *CaMet15NH* gene in pLate52 expression vector (Thermo Scientific, MA, USA). The antifungal *in vitro* activity was tested on *Candida albicans* ATCC 10231, *Candida glabrata* ATCC 90030, *Candida krusei* ATCC 6258, *Candida parapsilosis* ATCC 22019, *Candida tropicalis* DSM 11953, and *Candida dubliniensis* CBS 7987.

Two mutant *C. albicans* strains, namely CAI4 u*ra3Δ::imm434/ura3Δ::imm434*^17^ and 128 CAI4 *ura3D::imm434/ura3D::imm434 mup1D::hisG/mup1D::hisG* + pCaEXmpd^18^ were used in studies on influence of methionine permease on BIA antifungal activity. The latter strain was a generous gift from Prof. Patrick van Dijck, KU Leuven, Belgium.

*E. coli* cells were cultured on solid and in liquid Luria-Bertani (LB) media (0.5% yeast extract; 1.0% peptone; 1.0% NaCl; ampicillin 100 µg/mL). Yeast strains were grown on solid and in liquid YPG medium (1% yeast extract; 1% peptone; 2% glucose). The solid versions of LB and YPG media contained 1.5% agar.

#### Antifungal in vitro activity determination

Yeast cells were cultured on YPG (Yeast extract-Peptone-Glucose) solid medium overnight. The picked colonies were used as inocula for Minimal Inhibitory Concentration (MIC) determination by a serial two-fold dilution method in the 96-well microplate format in RPMI-1640 medium, according to the M27-A3 procedure^19^ Alternatively, the MIC values were determined by the same method in a minimal YNB w/o amino acids + 2% glucose medium, containing ammonium sulphate, 1 g/L (YNB AS) or L-glutamate, 0.5 g/L (YNB SG) as a nitrogen source. If necessary, the medium was supplemented with L-methionine at required concentration. The inoculum size was ∼10^4^ colony-forming units (CFU)/mL. Microplates were incubated at 37 °C for 24 h. The optical density of cell suspensions in individual wells was measured at 600 nm with a microplate reader (TECAN Spark 10 M, Austria). The MIC_90_ and MIC_50_ parameters are defined as the lowest concentration of an antifungal compound that inhibited growth by at least 90% or 50%, respectively, comparing to the drug-free control. All measurements were performed in triplicate.

### Checkerboard dilution assay

Antifungal *in vitro* activity of two agents in combination was quantified by the checkerboard technique in 96-well microplate format in YNB AS medium. Serial two-fold dilutions of both agents were prepared, so that each well contained a unique combination of concentrations of two antifungal agents. The concentrations were chosen to encompass the MIC_50_ values of both agents tested in a particular experiment. Solutions in wells were inoculated with 10^4^ CFU/mL of *C. albicans* ATCC 10231 cells from the overnight culture. After 24 h incubation at 37 °C, the optical density of cell suspensions in individual wells was measured at 600 nm with a microplate reader. All measurements were performed in triplicate. The fractional inhibitory concentration index (FICI) was calculated according to the formula:

$$\text{FICI = }\frac{{\text{MIC}}_{50}\text{ of agent }1\text{ in combination}}{{\text{MIC}}_{50}\text{ of agent }1\text{ alone}}\text{ + }\frac{{\text{MIC}}_{50}\text{ of agent }2\text{ in combination}}{{\text{MIC}}_{50}\text{ of agent }2\text{ alone}}$$

The calculated FICI values were interpreted according to the recommendations given by Odds^20^, where synergy is defined by FICI ≤ 0.5, indifferent effect is for 0.5 < FICI ≤ 4 and antagonistic interaction is for FICI >4. The synergy score was also visualised and analysed with the help of Combenefit software (version 2.021, Cancer Research UK Cambridge Institute, UK)^21^ using the Loewe additivity model.

#### Enzyme isolation and purification

In enzymatic studies, the previously prepared preparations of CaMet2NHp^10^ and CaMet15NHp^22^ and CaStr2NHp, prepared as shown below, were used.

#### CaSTR2 cloning and expression

The orf19.5645 sequence, coding for putative *O*-acetyl-L-homoserine sulfhydrylase, retrieved from the Candida Genome Database, was amplified by PCR using the *Candida albicans* SC5314 genome as a matrix. 5′-AGAAGGAGATATAACTATGCCTTCACAAGAAATAGGGAT**-3′** and 5′-GGAGATGGGAAGTCATTATATACTTTCATCTAAACTTTTTTGC**-3′** oligonucleotides were used as forward and reverse primers respectively. The obtained PCR product was cloned into pLate52 vector containing sequence coding for the N-terminal hexaHis tag, using an aLICator Ligation Independent Cloning and Expression System (Thermo Scientific, MA, USA). The obtained plasmid was verified by nucleotide sequencing. *Escherichia coli* ER2566 cells transformed with pLate52:CaSTR2NH were inoculated into LB broth supplemented with 100 µg/mL ampicillin and left to grow overnight at 30 °C. The starter culture (10 ml) was added to 800 ml LB broth supplemented with ampicillin and incubated at 30 °C (200 rpm) until the OD_600_ reached 1.0. Isopropyl-β-D-thiogalactoside (IPTG) was added to the final concentration of 1 mM to induce protein expression, after which the cell suspension was incubated for 24 h at 15 °C (200 rpm) before cell harvesting.

#### CaStr2NHp purification

The cell pellet was resuspended in buffer A (20 mM Tris-HCl buffer pH 8.0; 5 mM imidazole; 500 mM NaCl; 1 mM Tween 20) supplemented with cOmplete^™^ Protease Inhibitor Cocktail (Hoffmann-La Roche, Switzerland) and 10 mM dithiothreitol (DTT), and sonicated on ice, followed by centrifugation at 10,000 rpm for 20 min. The resulting supernatant was subjected to affinity chromatography with a 5 ml HisTrap^™^ Fast Flow (Cytiva, MA, USA) column in the KTA Pure^™^ chromatography system (Cytiva, MA, USA). The column was equilibrated with buffer A before sample injection, and the target protein, *i.e.,* CaStr2NHp, was eluted with buffer B (20 mM Tris-HCl buffer pH 8.0; 500 mM imidazole; 500 mM NaCl; 1 mM Tween 20). Fractions containing the target protein were concentrated to a final volume of 5 ml and loaded onto Superdex^™^ 200 16/600 GL column (Cytiva). The isocratic elution was carried out with 25 mM Tris-HCl buffer, pH 8.0, containing 0.3 M NaCl.

Purification progress was monitored by SDS-PAGE (18 V cm^−1^, 5% stacking gel and 10% resolving gel) and protein molecular mass was determined by NATIVE-PAGE (NativePAGE^™^ Novex Bis-Tris Gel System kit/Invitrogen/, according to the given manual).

#### Enzymatic activity measurements

The Met2p activity was measured according to the method of Foyn et al.^23^, based on quantification of CoA-SH formed from the AcCoA substrate, using the 5,5′-dithio-bis-(2-nitrobenzoic acid) (DTNB). The reaction mixture was composed of 3.5 mM AcCoA lithium salt, 10 mM L-Hse and 0–10 mM inhibitor in 50 mM sodium phosphate buffer, pH 8.0, containing 100 mM NaCl and 1 mM EDTA, in a total volume of 50 µL. The reaction was started by an addition of the enzyme (30 nM) and carried out for 5 min at 37 °C. The reaction was stopped by the addition of 100 µL of quenching buffer (3.2 M guanidinium-HCl in 100 mM sodium phosphate buffer, pH 5.8) and then 20 µL of DTNB buffer (10 mg/mL DTNB and 10 mM EDTA in 100 mM sodium phosphate buffer, pH 6.8) was added. Aliquot of the mixture (150 µL) was transferred to microplate wells and absorbance was measured spectrophotometrically at 412 nm with a microplate reader.

The Met15p activity was quantified by determination of L-Hcy formed using the HPLC-DAD-MS method with a pre-column derivatization of L-Hcy with DTNB. The reaction mixture contained 25 mM Tris-HCl pH 8.0, 100 mM NaCl, 10 mM L-Hse; 5 mM Na_2_S, 0.2 mM PLP and 0 – 10 mM inhibitor in a total volume of 50 µL. The reaction was started by an addition of the purified enzyme (10 nM) and run for 10 min at 37 °C. Samples of 10 µL were collected at time intervals, reaction was terminated by an addition of 100 µL of the stopping buffer (100 mM sodium phosphate, 3.2 M guanidinium-HCl, pH 5.8), followed by an addition of 20 µL of DTNB buffer. Aliquots of 10 µL were injected into the HPLC-DAD-MS system (Zorbax Eclipse C18 column 5 µm, 250 mm x 4.6 mm (Agilent, CA, USA); mobile phase A: 0.45% HCOOH, B: ACN, flow rate 1.2 ml/min). Derivatized L-Hcy was detected at 330 nm.

For determination of kinetic parameters of CaMet15NHp inhibition by BIA, the reaction mixtures contained a constant concentrations of Na_2_S (1 mM) and PLP (0.2 mM) and variable concentrations of L-OAH (0–14 mM) and BIA (0–2.5 mM). K_i_ was calculated with GraphPad Prism 8.0 (GraphPad Software Inc., CA, USA).

The Str2p activity was measured using a HPLC-DAD-MS method, based on quantification of decrease in L-Cys (one of the substrates) concentration, pre-column derivatized with DTNB. The reaction mixture contained 5 mM L-Cys, 10 mM L-OAH, 0.2 mM PLP, 0–10 mM inhibitor, and CaStr2NHp (10 nM) in Str2p buffer (25 mM Tris-HCl pH 7.5, 100 mM NaCl, and 2 mM EDTA), in a final volume of 50 μL. The reaction was started by addition of the enzyme and carried out for 10 min at 37 °C, then stopped by the addition of 100 μL of STOP buffer (3.2 M guanidinium-HCl in 100 mM sodium phosphate dibasic, pH 6.8). Next, 20 μL of DTNB buffer (100 mM sodium phosphate dibasic pH 6.8, 10 mM EDTA, 10 mg/mL DTNB) was added, and 10 μL aliquot was injected into the HPLC-DAD Zorbax Eclipse C_18_ column (5 μm, 250 mm x 4.6 mm). Signals derived from the L-Cys-TNB conjugate were detected at 330 nm.

For determination of kinetic parameters of CaStr2NHp inhibition by BIA, the reaction mixtures contained a constant concentrations of L-Cys (5 mM) and PLP (0.2 mM) and variable concentrations of L-OAH (0–20 mM) and BIA (0–4 mM). K_i_ was calculated with GraphPad Prism 8.0 (GraphPad Software Inc., CA, USA).

### Molecular modelling

A putative structure of CaMet15p and CaStr2p receptors for docking calculations was prepared based on the AlphaFold^24^ model, generated for the amino acid sequences of these proteins and the known structures of *S. cerevisiae* Met15p (8OVH) and *Lactobacillus plantarum* Str2p (9VSM) as a matrixes. A dimeric form of the Met15p protein was used as a receptor, since the single binding site is formed by residues belonging to the two distinct chains. The placement and conformation of the PLP molecule present in the binding site were also modelled based on its conformations in matrix enzymes and fixed as a part of the receptor during all docking simulations.

Since the reaction catalysed by the enzyme involves the formation of a Schiff base by α-amino group of a ligand with the PLP molecule, besides the standard semi-flexible docking protocol, an additional covalent docking was performed for each ligand, with its α-amino moiety bound to PLP. To achieve this, an additional interaction map was created with a Gaussian potential centred on the position of the bound nitrogen with width δ and amplitude ε parameters set to 3 Å and 10 kcal/mol respectively.

Structures of ligands were first built using HyperChem software [HyperChem(TM) Professional 8.0, Hypercube, Inc., 1115 NW 4th Street, Gainesville, Florida 32601, USA], and then converted to PDBQT format with AutoDockTools scripts^25^. The docking grid was centred on the PLP aldehyde moiety and its size was set to 35 Å in each direction. Fifty docking simulations were performed for each ligand using the Autodock 4.2 and the LGA algorithm^25^. The results were clustered using a cluster tolerance threshold of 2.0 Å, and low-energy poses from the most populated clusters were selected for further analysis.

All figures presenting the results of molecular modelling were prepared with the VMD software^26^.

## Results and discussion

### Chemistry

#### Synthesis of BIA

BIA was synthesised in a six-step procedure, the course of which is shown in Scheme 2, starting from the commercially available Boc-β-chloro-L-alanine. The method used was a slightly modified version of the original protocol of Baldwin et al.^27^ Synthesis started with a coupling reaction between *N*-(tert-butoxycarbonyl)-β-chloro-L-alanine and *N*-Boc protected hydroxylamine, in the presence of 1-ethyl-3–(3-dimethylaminopropyl)carbodiimide (EDCI), resulting in compound **1**. Treatment with NaH gave a cyclic product **2**, which was subsequently opened under the action of water and caesium carbonate. The obtained compound **3** was then converted into diphenylmethyl ester **4** upon reaction with diphenyldiazomethane. The ester **4** was coupled with propiolic acid to yield **5**. All protections were removed upon reflux in formic acid, and cyclisation occurred, affording finally BIA. The cumulative yield of the six-step synthesis was 18%. Purity of the final product was 97.4% (Supplementary materials, S2 and its identity was confirmed by NMR and HRMS analysis (Supplementary materials, S1-2).

**Scheme 2.**
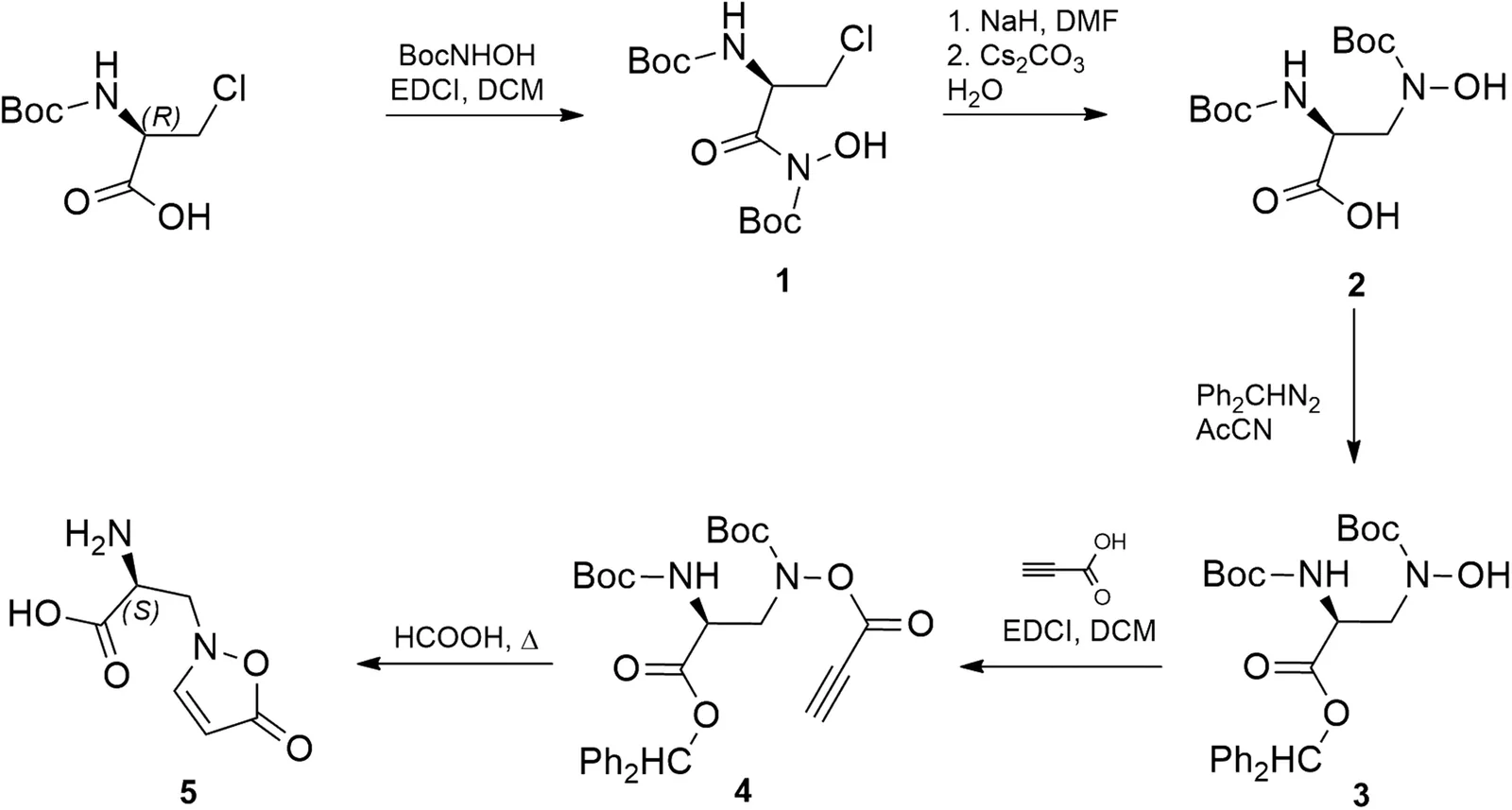
Synthesis of BIA. Boc, *t*-butoxycarbonyl; DMF, dimethylformamide; MeCN, acetonitrile; EDCI, 1-ethyl-3–(3-dimethylaminopropyl)carbodiimide; DCM, dichloromethane

### Biological properties

#### Anticandidal in vitro activity of BIA

BIA was previously reported to exhibit growth inhibitory effect against *S. cerevisiae* and phytopathogenic fungi^13^^,^^14^ but its activity against human fungal pathogens has not been investigated so far.

#### BIA exhibits anticandidal in vitro activity and its growth inhibitory action is reversed upon L-met supplementation

Anticandidal *in vitro* activity of BIA was tested against six reference strains of human pathogenic yeasts of the Candida genus. The MIC_50_ and MIC_90_ values were determined by the serial dilution method in the 96-well microplate format, in three growth media, namely two minimal YNB media containing ammonium sulphate or sodium glutamate as a nitrogen source and the RPMI-1640 medium. The MIC values were also determined in YNB media supplemented with L-methionine, 5 mM. Conditions of the assay in YNB and RPMI 1640 media were the same as outlined in the CLSI recommendations^19^. Results are presented in Table 1.

**Table 1.** MIC_90_ and MIC_50_ values of BIA against reference strains of *Candida* spp., determined in YNB AS, YNB SG and RPMI-1640 media. The given values correspond to three consistent results from independent experiments.

MIC_90_ (MIC_50_) [µg/mL]					
	Growth medium				
Strain	YNB AS	YNB AS + L-Met 5 mM	YNB SG	YNB SG + L-Met 5 mM	RPMI-1640
*C. albicans*	2 (1)	>256 (>256)	2 (1)	>256 (256)	256 (128)
*C. glabrata*	4 (1)	>256 (>256)	2 (1)	>256 (128)	256 (128)
*C. krusei*	128 (64)	>256 (>256)	64 (32)	>256 (>256)	>256 (>256)
*C. parapsilosis*	4 (1)	>256 (>256)	4 (2)	>256 (>256)	>256 (256)
*C. tropicalis*	8 (2)	>256 (>256)	8 (4)	>256 (>256)	>256 (>256)
*C. dubliniensis*	0.5 (0.25)	>256 (>256)	1 (0.5)	>256 (>256)	>256 (>256)

BIA demonstrated good antifungal activity in YNB media against *C. albicans, C. glabrata, C. parapsilosis, C. tropicalis*, and *C. dubliniensis*, with MIC_50_ values in the 0.25 − 4 µg/mL range and MIC_90_ equal to 0.5 – 8 µg/mL. *C. krusei* appeared much less susceptible (MICs in the 32 – 128 µg/mL range).

Presence of L-Met, 5 mM, in YNB media abolished BIA growth inhibitory activity. Very poor (*C. albicans* and *C. glabrata*), or no (other Candida spp.) activity of BIA was noted in RPMI-1640 medium. It is worth mentioning therefore, that this medium contains L-Met, 15 µg/mL (∼0.1 mM). The observed lack of the antifungal activity of BIA, as a result of the presence of L-Met in the culture medium, can be explained at least in two ways: as a consequence of providing fungal cells with an amino acid whose biosynthesis was inhibited by BIA or as a result of competition between BIA and L-Met for the binding site in the transmembrane transport protein, preventing intracellular accumulation of BIA.

To verify both hypotheses, we compared growth inhibitory activity of BIA against *C. albicans* CAI4 strain and CAI4-derived 128 CAI4 mutant cells lacking the L-Met-specific permease Mup1p and effects of presence of L-Met or L-Hcy in the growth medium on MIC values.

As shown in Table 2, MICs of BIA against CAI4 cells were the same as those against ATCC 10231 cells. Presence of L-Met at 0.1 mM resulted in the substantial increase of MICs against the CAI4 cells, notably to the values found in RPMI-1640 medium against ATCC 10231 cells (Table 1). A marked enhancement of MICs was also observed when YNB medium was supplemented with L-Hcy, 0.1 mM. L-Met at 50 µM had a lower effect and at 25 µM, the MIC values were only two-fold higher than those determined in minimal medium without L-Met supplementation. It is noteworthy therefore, that Suliman et al. found that 100 µM L-Met rescued methionine auxotrophy of *C. albicans* cells due to the deletion of the *MET6* gene coding for methionine synthase, *i.e.,* enzyme catalysing the final step of MBP^8^. However, in our hands, some reversal of growth inhibitory effect of BIA action was observed also at 25–50 µM L-Met but not by L-Hcy, 25 µM. Effective reversal of the growth inhibitory effect of BIA by L-Met was also observed for *C. glabrata* cells, where 15 µM L-Met was sufficient for ∼40% reversal and 125 µM for more than 90% reduction of growth inhibition by BIA, 64 µg/mL (Figure 2).

**Figure 2.**
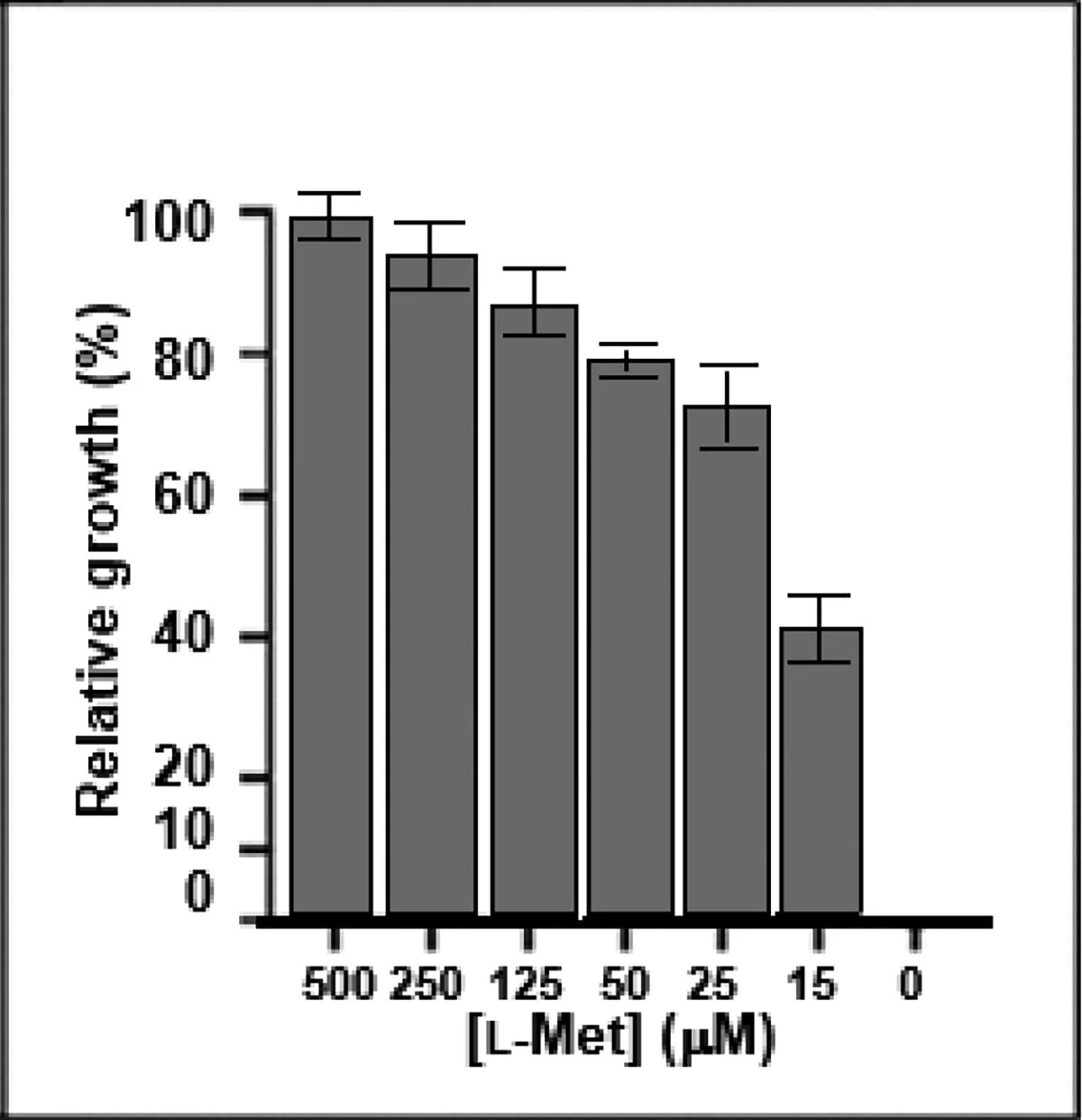
Reversal by L-Met of BIA-induced *C. glabrata* growth inhibition. The experiment was performed in YNB AS medium supplemented with L-Met in 0 – 0.5 mM concentration range. BIA was present at 64 µg mL^−1^. The error bars represent ± SD.

**Table 2.** MIC_90_ and MIC_50_ values of BIA against *C. albicans* CAI4 and 128 CAI4 strains. The given values correspond to three consistent results from independent experiments.

MIC_90_ (MIC_50_) [µg/mL]						
	Growth medium					
	YNB AS	YNB AS + L-Met 100 µM	YNB AS + L-Met 50 µM	YNB AS + L-Met 25 µM	YNB AS + L-Hcy 100 µM	YNB AS + L-Hcy 25 µM
CAI4	2 (1)	256 (128)	16 (8)	4 (2)	128 (64)	2 (1)
128 CAI4	>256	>256	>256	>256	>256	>256

#### Antifungal effect of BIA and L-PEN in combination is stronger than that of each agent alone

In our previous studies, L-penicillamine (L-PEN), demonstrating antifungal *in vitro* activity, was found to be an inhibitor of CaMet2p, competitive in respect to L-Hse, with IC_50_ = ∼2.5 mM. The growth inhibitory effect of L-PEN was completely abolished when growth medium was supplemented with L-Met, thus indicating that CaMet2p is the only essential target of this compound in fungal cells^10^. This finding was surprising, because L-PEN has been known so far as a non-specific inhibitor of several PLP-dependent enzymes, by forming PLP-thiazolidine adducts^28^, while Met2p does not employ the PLP cofactor. Interestingly, in our hands L-PEN appeared a poor inhibitor of PLP-dependent CaMet15p^22^ and CaStr2p (this work, details not shown), with IC_50_ values well above 10 mM.

A synergistic growth inhibitory effect of compounds targeting two enzymes catalysing different steps of the same biosynthetic pathway is a phenomenon known as a sequential blockade. The antibacterial effect of sulfamethoxazole and trimethoprim in combination inhibiting two enzymes of the folate biosynthesis pathway is a well-known classic example. In this study, we checked the possibility, whether L-PEN and BIA, *i.e.,* inhibitors of enzymes catalysing two consecutive steps of MBP, may act synergistically. The checkerboard dilution assay in 96-well microplate format in YNB AS medium was used. Results of this assay, presented as heat maps in Figure 3, indicate a slight synergistic affect. Actually, the MIC_50_ values of L-PEN and BIA in combination (64 µg/mL and 0.25 µg/mL, respectively) were lower than these of each compound alone (256 µg/mL and 1 µg/mL, respectively). The calculated FICI is 0.5, which is the upper border value for synergy^20^ so that the practical significance of L-PEN:BIA interaction is marginal. Nevertheless, to our best knowledge, this interaction is the only known example of synergistic action of antifungals targeting two enzymes of the same biosynthetic pathway.

**Figure 3.**
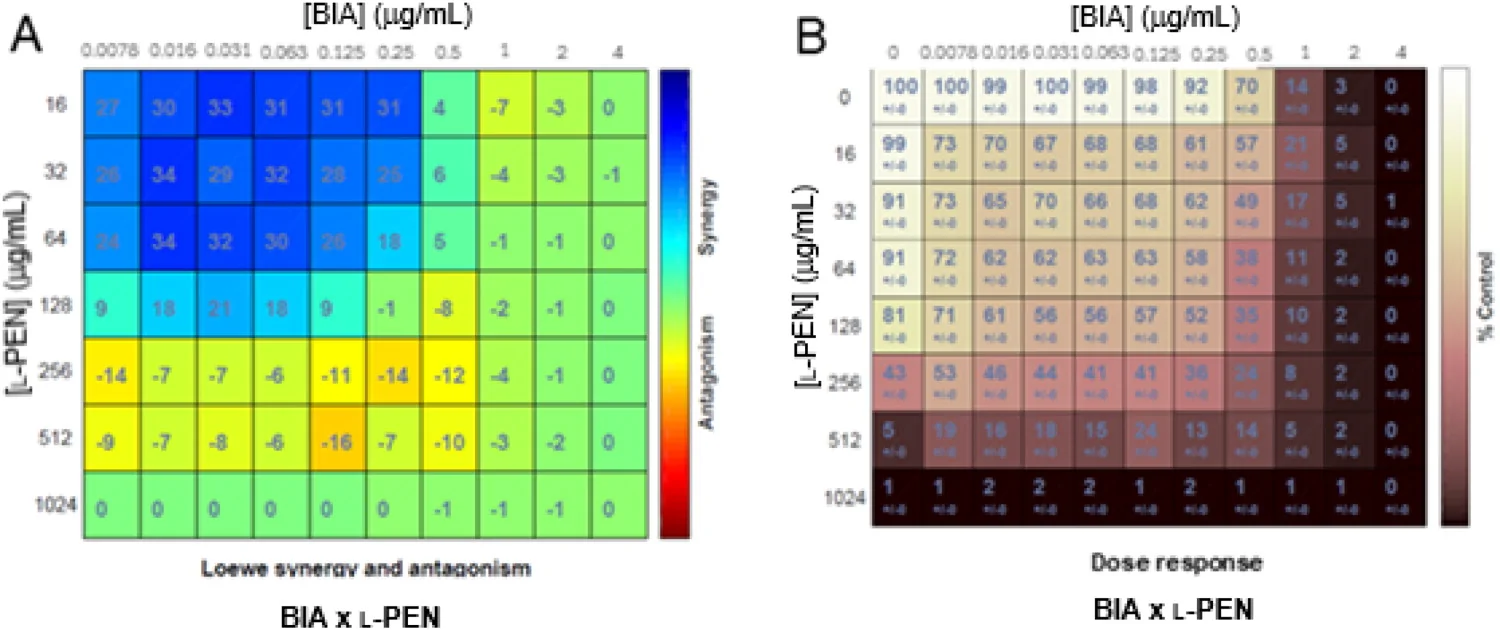
A. Generated heat map of results of the checkerboard dilution test on interaction between BIA and L-PEN. Blue, synergistic effect; red, antagonistic effect; green, indifferent effect. B. Generated heat map of results of the checkerboard dilution test demonstrating growth inhibition compared to the untreated control. Interaction effect scores were obtained using the Loewe additivity model.

#### BIA takes advantage of the Mup1p methionine permease as a route of entry into C. albicans cells

The observed apparent resistance of 128 CAI4 cells (MIC > 256 µg/mL, Table 2) to BIA, provides a rational explanation for the observed changes in *C. albicans* sensitivity to BIA action, depending on L-Met concentration. The only difference between CAI4 and 128 CAI4 cells is a lack of the *MUP1* gene coding for methionine permease in the latter^18^. This finding suggests that BIA is most likely transported to *C. albicans* by Mup1p. The apparent K_M_ for L-Met of *C. albicans* Mup1p is as low as 28 µM^18^, while that for BIA as a non-canonical substrate, is possibly much higher. One may thus expect that L-Met at 0.1–0.05 mM should effectively prevent BIA binding to Mup1p. On the other hand, reversal of the BIA-induced growth inhibitory effect by L-Hcy, 0.1 mM but not by L-Hcy, 25 µM, can be explained as a consequence of providing *C. albicans* cells with the substrate for Met6p, in an amount sufficient to meet the cellular demand for methionine. Strong growth inhibitory effect of BIA against CAI4 cells in YNB supplemented with 25 µM L-Hcy and insensitivity of 128 CAI4 suggests lack of competition between BIA and L-Hcy for the uptake by Mup1p.

### Enzyme inhibition

#### Construction, purification and characterisation of recombinant CaStr2NHp

The *CaSTR2* gene, cloned into pLate52 vector introducing sequence coding for N-terminal hexaHis-tag, was expressed in *E. coli* ER2566 cells and the gene product, *i.e.,* CaStr2NHp, was purified by using oligoHis-directed affinity chromatography, The SDS-PAGE analysis of cell-free extract from ER2566 cells transformed with pLate52::CaSTR2 and preparation obtained after affinity chromatography showed that purity of the final preparation was 79% (Supplementary materials, S2). A further purification, up to the 98% level, was obtained by size exclusion chromatography (Supplementary materials, S4). A molecular mass of the isolated CaStr2NHp, determined by SDS-PAGE, was 67.5 ± 1.4 kDa, in good agreement with the 68.04 kDa value calculated from the amino acid sequence. The native-PAGE analysis revealed presence of a dominant monomeric form and a dimeric form as a minor component of CaStr2NHp preparation (Supplementary materials, S3).

#### BIA is an inhibitor of CaMet15p and CaStr2p

Reversal of the growth inhibitory effect of BIA by L-Met and L-Hcy clearly suggested interference of this agent in MBP, most likely by inhibition of Met2p, Met15p and/or Str2p. Having in our hands the recombinant, *N*-oligoHis-tagged versions of these enzymes from *Candida albicans* we were able to test the inhibitory potential of BIA against them. In the preliminary screening study we determined the effect of 10 mM BIA on activity of three enzymes. As shown in Figure 4, BIA at 10 mM strongly inhibited CaMet15NHp and CaStr2NHp activity (87.5% and 81.1% inhibition, respectively), while activity of CaMet2NHp was almost not affected (∼6% inhibition).

**Figure 4.**
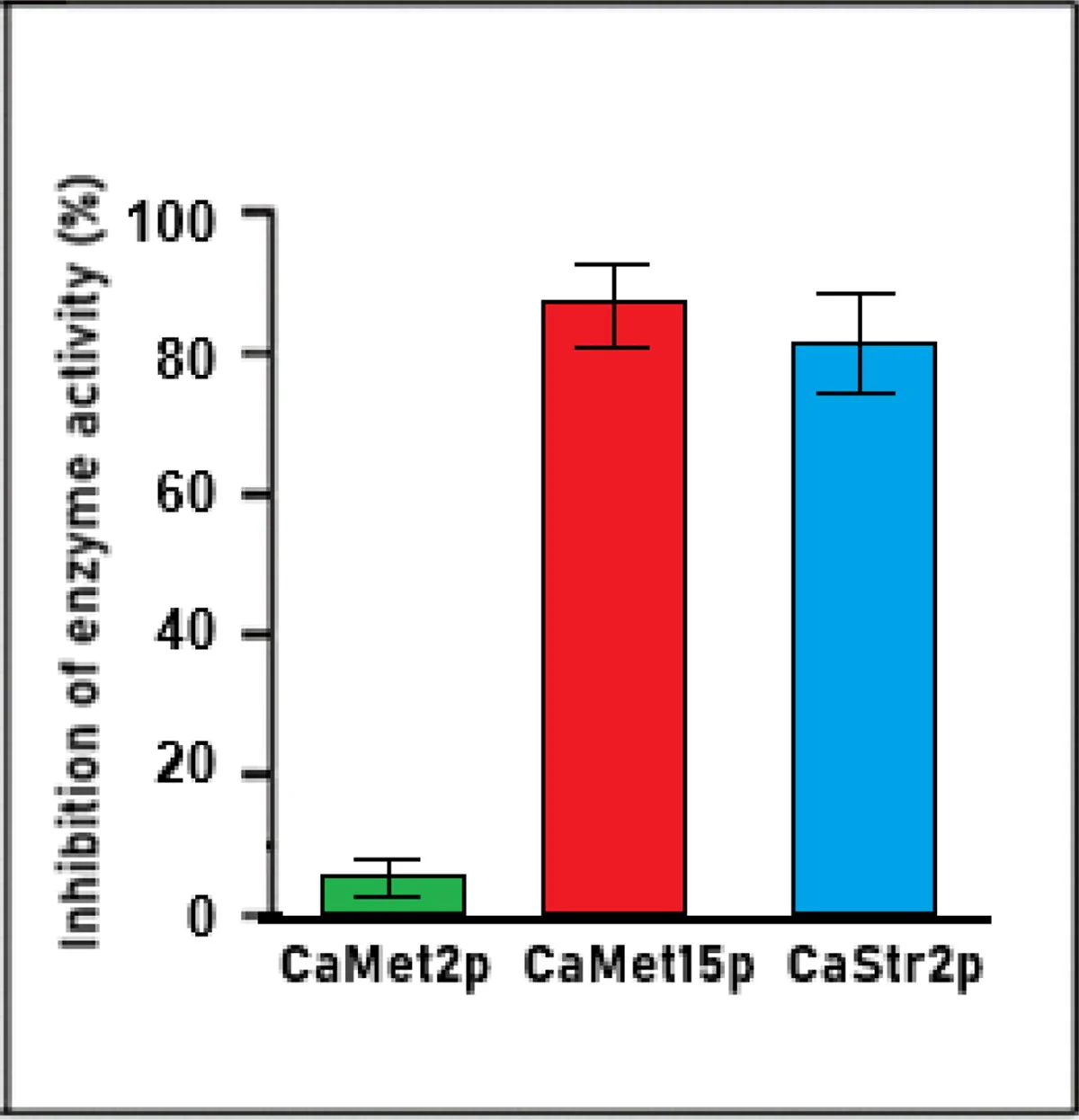
Inhibitory potency of BIA (10 mM) against three *C. albicans* enzymes of MBP. The values represent percentage inhibition relative to enzyme activity measured in the absence of BIA. Data shown are the mean values of three independent experiments (± SD).

In the more detailed investigation of CaMet15NHp inhibition by BIA, the IC_50_ value of 1.05 mM was determined (Figure 5).

**Figure 5.**
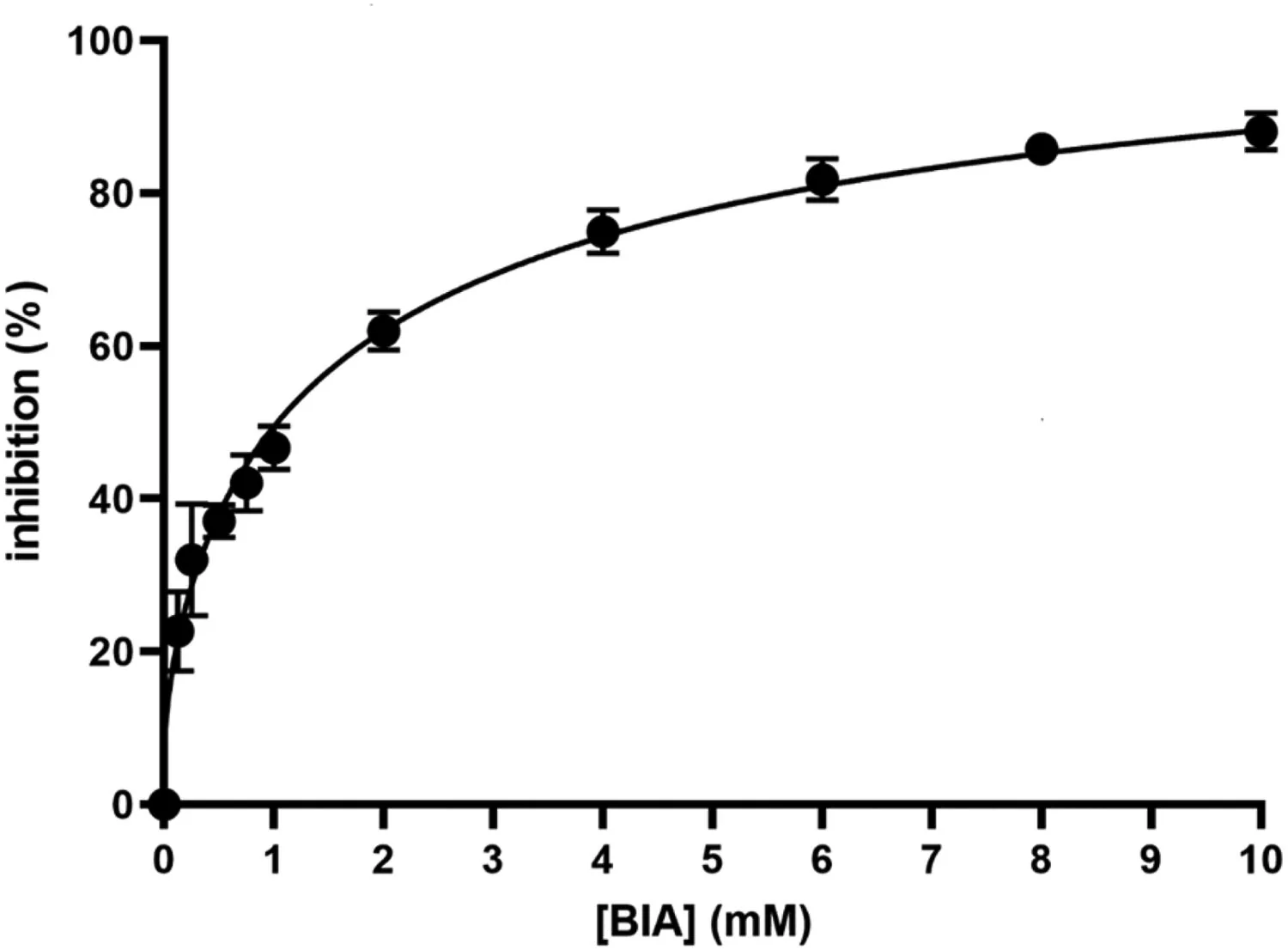
Inhibition of CaMet15NHp activity by BIA at fixed L-OAH and Na_2_S concentrations. The error bars represent ± SD.

The pattern of Lineweaver-Burk plot for enzyme inhibition in respect to L-OAH concentration, presented in Figure 6, unequivocally indicates its competitive character. The K_i_ value for this inhibition was 0.711 mM. This value is almost 13 times lower than the previously determined K_M_ = 9.12 mM of this enzyme for L-OAH^22^. It should be noted that in the only previous studies on fungal Met15p from fission yeasts *Schizosaccharomyces pombe,* the K_M_ value for L-OAH equal to 12.5 mM was determined^29^, so that it seems that the K_M_ values of fungal Met15p versions for L-OAH are about an order of magnitude higher than those of their bacterial counterparts (0.6–1.0 mM)^30–32^. The intracellular L-OAH level is not known. However, assuming that most likely it should be close to the K_M_ value for this substrate, it is obvious that BIA is able to effectively compete with L-OAH for binding at the fungal Met15p active centre.

**Figure 6.**
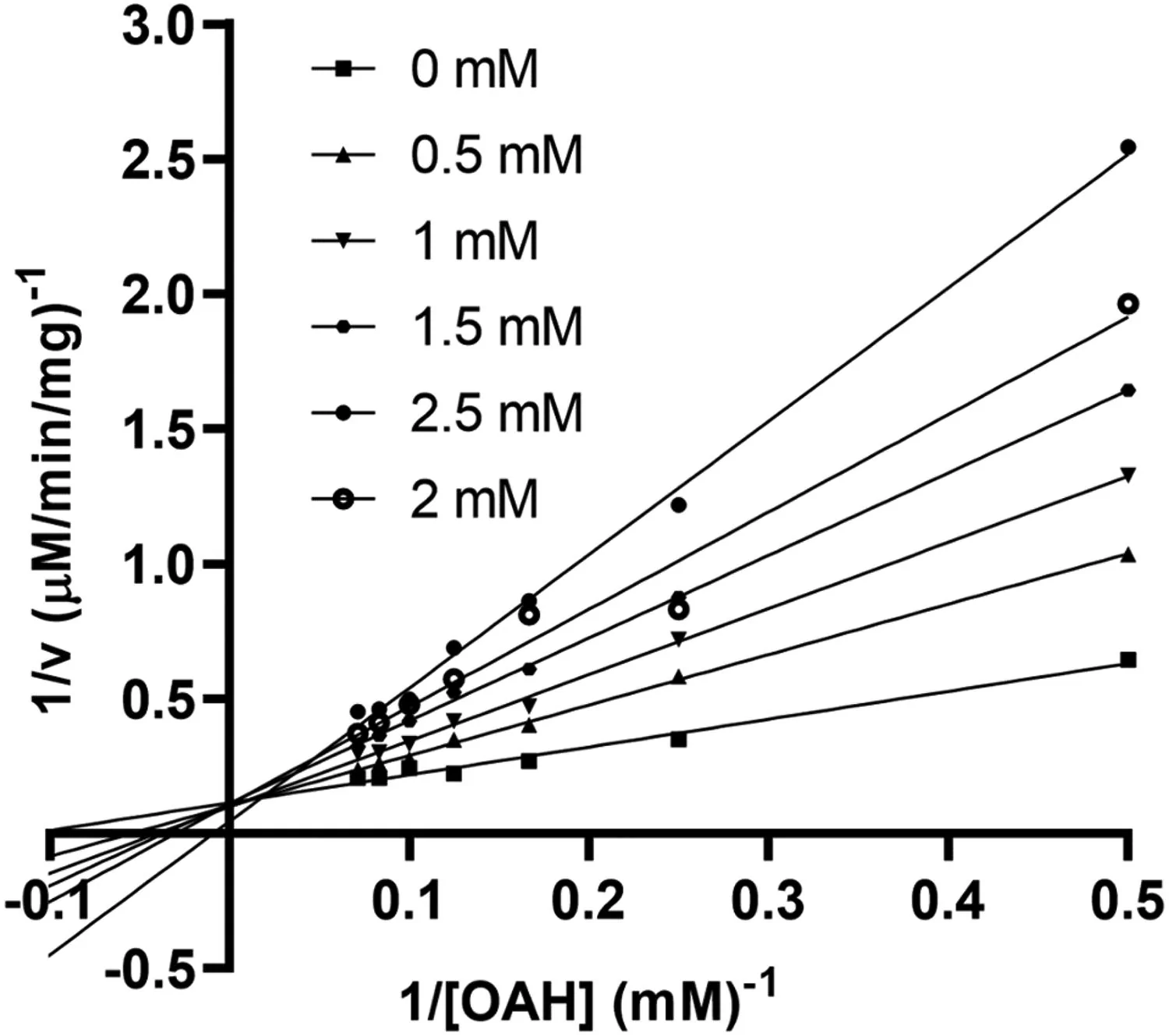
Competitive inhibition of CaMet15NHp by BIA in respect to L-OAH. BIA concentrations are indicated inside.

It was previously reported that BIA did not exhibit any antibacterial effect^14^, despite the fact that bacteria possess MBP employing Met15p as an enzyme catalysing L-Hcy formation. However, a bacterial version of Met15p uses most often *O*-succinyl-L-homoserine, not L-OAH^33^. In consequence, BIA, as a L-OAH analogue, may not bind effectively at bacterial Met15p active centre.

Reversal of the growth inhibitory effect of BIA by L-Met and L-Hcy clearly suggests that enzymes employing L-OAH as a substrate, *i.e.,* Met15p and Str2p, are the only targets for this compound in *C. albicans* cells. Therefore, the fungistatic effect of BIA action on *C. albicans* is most likely a consequence of inhibition of these enzymes. From this point of view, more than one hundred lower value of MIC_50_ (1 µg/mL, *i.e.,* ∼0.0058 mM), compared to IC_50_ for CaMet15p (1.05 mM) may seem surprising. However, an active transport of BIA by the proton gradient dependent Mup1p permease most probably leads to the intracellular accumulation of this agent against the concentration gradient, possibly even up to milimolar concentrations. Although we were not able to confirm this assumption experimentally, due to the lack of isotopically labelled BIA, analogy with documented examples of uptake and accumulation of other amino acid antimicrobials makes it very likely. Particularly, Ziegelbauer at al., reported more than 200-fold accumulation of an amino acid antifungal agent icofungipen in *C. albicans* cells, due to its transport by the H^+^-coupled branched-chain amino acid transporter^34^.

### In silico analysis of enzyme:inhibitor interactions

#### BIA binding at CaMet15p active Centre and its hypothetical action as a dead-end inhibitor

Looking for the molecular basis of CaMet15p inhibition by BIA, competitive in respect to L-OAH, a series of *in silico* docking experiments were performed. A putative structure of CaMet15p was generated by AlphaFold, using the X-ray structure of Met15p from *S. cerevisiae* (PDBID: 8OVH) as a template. The dimeric form of the Met15p protein was used as the receptor, since the single binding site is formed by residues belonging to the two distinct chains. The obtained modelled structure of CaMet15p homodimer is presented in Figure 7(A). Regular docking of L-OAH and BIA to the active centre of this receptor, followed by covalent docking with the α-amino group of each ligand bound to the PLP cofactor was carried out, using the AutoDock 4.2 and the Lamarckian Genetic Algorithm (LGA). Structures of the enzyme:ligand complexes thus obtained are shown in Figures 7(B) and 7(C).

**Figure 7.**
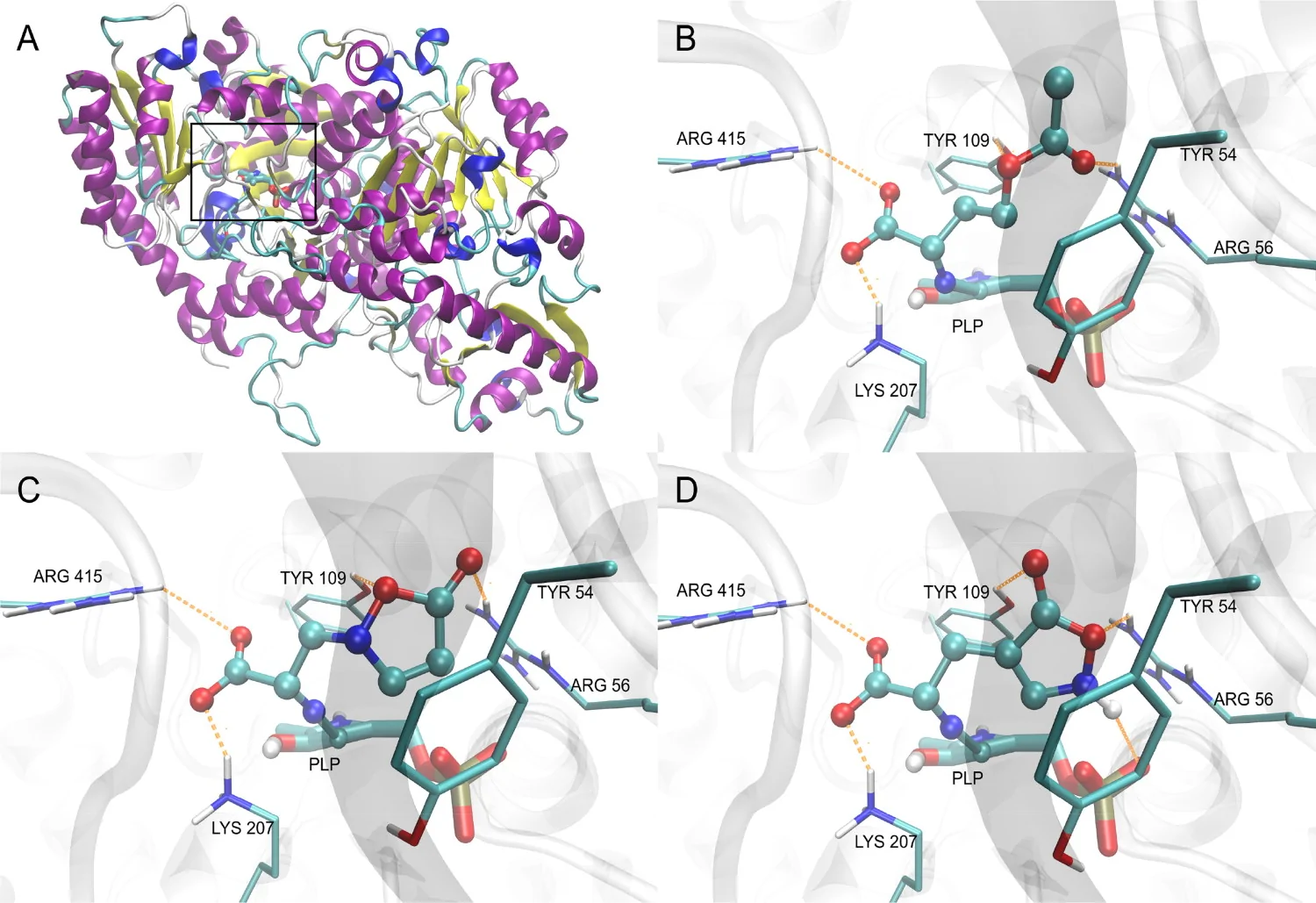
A. Structure of the homology modelled CaMet15p with PLP in the binding site (marked with rectangle). Results of covalent docking of L-OAH (B), BIA (C) and TAN-950 A (D). Essential residues are drawn with thin sticks and PLP as a thicker sticks model. Ligands are depicted as ball and sticks models.

In CaMet15p:L-OAH complex, the substrate is oriented in such a way that its acyl group is positioned proximal to the catalytic residues, enabling the expected γ-elimination of acetate, as proposed by Tran et al.^35^ and Kulikova et.al^36^.

Results of the docking simulations of BIA indicate that the heterocyclic ring of this ligand occupies a position closely resembling the position of the acyl moiety of L-OAH, with the ring oxygen atom of BIA effectively mimicking the position of the ester oxygen of L-OAH, facilitating interaction with the active site environment, particularly the Tyr109 residue, in a manner analogous to the native substrate (Figure 7C). The carbonyl oxygen of BIA is engaged in stabilising interactions, particularly with the guanidinium moiety of Arg56, additionally anchoring the ligand in the catalytic pocket. It is clear therefore that BIA can compete with the natural CaMet15p substrate, *i.e.,* L-OAH, for binding in the CaMet15p active site. This is consistent with the experimentally evidenced competitive character of enzyme inhibition with respect to L-OAH, given that BIA can approximate the key binding determinants of L-OAH. The calculated binding energies of L-OAH and BIA were − 4.70 kcal/mol and − 5.75 kcal/mol, respectively. However, given the uncertainty associated with the AutoDock 4.2 scoring function, this difference should not be interpreted as quantitative evidence of a higher affinity of BIA for the CaMet15p. Rather, it further supports the hypothesis that both ligands may indeed bind competitively with comparable predicted strength.

Obviously, the docking results, particularly those obtained using homology modelled receptor structures, cannot be treated as experimentally validated binding modes and should instead be regarded as approximate models. However, the resulting geometries of the CaMet15p:L-OAH/BIA complexes are consistent with the active-site architecture inferred from homologous PLP-dependent enzymes, the conservation of key catalytic residues, and ligand conformations commonly observed in this structural context.

A putative course of reaction catalysed by bacterial Met15p, proposed by Tran et al.^35^ and Kulikova et al.,^36^ follows the general rules of the ping-pong mechanism, characteristic for the PLP-dependent enzymes employing amino acid substrates (Scheme 3A). In that proposal, L-OAH after binding and transimination with enzyme-bound PLP, undergoes two subsequent proton abstractions, followed by acetate elimination, preceded by the side chain oxygen atom protonation with the active site Tyr107 residue as a proton donor. Assuming that BIA can be bound at CaMet15p active centre as a L-OAH analogue and then forms imine with PLP, the initial steps of processing BIA should be the same as those of the natural substrate. Subsequent BIA oxygen ring protonation is very likely. In our docking simulations, the heterocyclic oxygen atom of BIA is located at a distance of 3.11 Å from the hydroxyl of Tyr109 residue, a counterpart of Tyr107 of bacterial Met15p, as shown in Figure 7(C). This is even shorter than a respective 3.58 Å distance of the oxygen atom of L-OAH from Tyr109 (Figure 7B). Therefore, protonation of BIA ring oxygen is facilitated and a subsequent breakage of the O-N bond is possible, as postulated in Scheme 3B. However, a further conversion of bound BIA is unlikely, due to the inability of acetate release and presence of the nitrogen atom, both precluding formation of an appropriate acceptor for S^2-^. It is therefore possible that BIA acts as a dead-end inhibitor of Met15p, i.e., it binds to the enzyme, preventing product formation without being consumed, creating a stable, non-productive („dead-end”) complex that blocks the active centre. This interpretation is supported by the docking pose of BIA, its close correspondence to the orientation of L-OAH in the modelled CaMet15p active site, and by analogy to ligand binding modes observed in homologous PLP-dependent enzymes, such as methionine γ-lyase (PDBID: 5M3Z)^37^. Nevertheless, the proposed dead-end character of CaMet15p inhibition by BIA should obviously be regarded as a mechanistic hypothesis based on structural modelling. Its definitive validation would still require experimental confirmation.

**Scheme 3.**
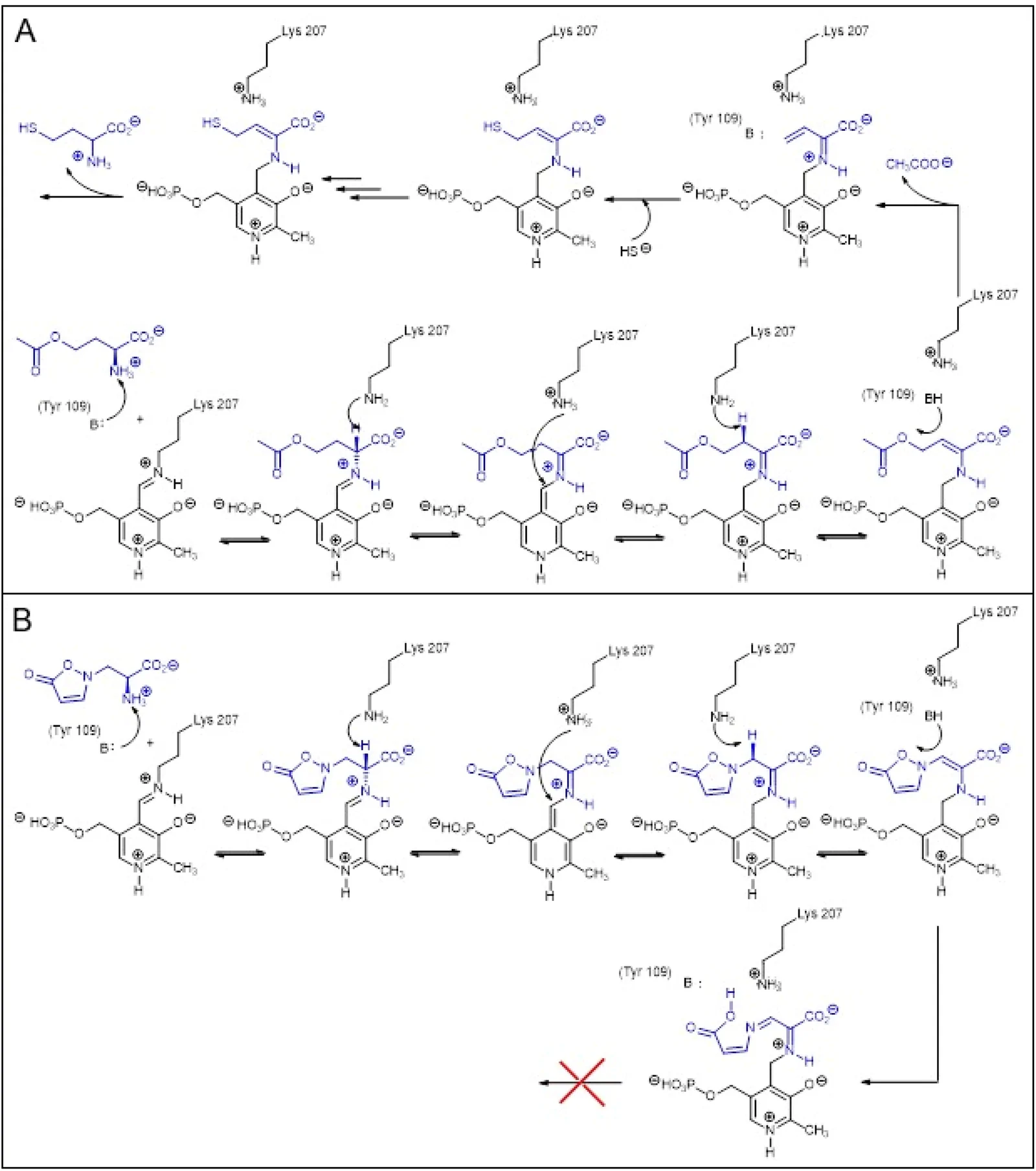
A. Putative course of γ-substitution reaction catalysed by L-homoserine *O*-acetyltransferase. B. Proposed mechanism of BIA processing at the active centre of CaMet15p.

In another docking experiment we investigated a possible arrangement of an antifungal antibiotic TAN-950 A^38^, a constitutional isomer of BIA (Figure 1), at CaMet15p active centre. The docking pose of TAN-950 A presented in Figure 7(D) reveals a pattern of interactions with essential binding site residues similar to that of BIA but with one significant difference. The positions of the ring oxygen and carbonyl carbon relative to BIA and L-OAH are swapped. This leads to a less optimal set of stabilising contacts, particularly with Tyr109 hydroxyl moiety, although formation of a hydrogen bond between this group and TAN-950 A carbonyl oxygen is possible. Notably, TAN-950 A does not contain a ring oxygen atom adjacent to the active site Tyr 109, so that an appropriate proton acceptor is missing. In consequence, although TAN-950 A could possibly bind in CaMet15p active centre at the L-OAH site, it is unlikely to behave as a dead-end inhibitor of the enzyme, due to the lack of an appropriate acceptor for Tyr109-derived proton.

The actual molecular target of TAN-950 A in fungal cells is unknown, but the results of its molecular docking to Met15p rule out its activity as an inhibitor of this enzyme, and consequently of MBP. This confirms the results of previous studies of TAN-950 A biological properties, which found that its growth-inhibiting effect is reversed not by L-Met but by L-Glu^38^. Therefore, it can be assumed that TAN-950 A acts as a glutamate analogue and its molecular target in fungal cells could be any essential enzyme having this amino acid as a ligand. Actually, the structural analogy between TAN-950 A and L-Glu was previously suggested, due to its activity as a glutamate agonist demonstrating affinity for three human excitatory amino acid receptors^39^.

#### The L-OAH binding site of CaStr2p is homologous to that of CaMet15p

The similar inhibitory potential of BIA towards CaMet15p and CaStr2p prompted us to perform molecular modelling of the latter enzyme. Since no crystal structure of fungal cystathionine γ-synthase is known, the molecular modelling of CaStr2p structure was based on available *Lactobacillus plantarum* version of this enzyme (PDBID: 9VSM)^40^ as a matrix. Notably, bacterial cystathionine γ-synthases are in general known to use *O*-succinyl-L-homoserine as a substrate^33^ but the enzyme from *Lactobacillus plantarum* was found to accept L-OAH^40^.

The comparative analysis of the spatial arrangement of key catalytic residues at the L-OAH binding site in the active centres of CaStr2p and CaMet15p models is presented in Figure 8. All essential residues identified at the L-OAH binding centre of CaMet15p, i.e., Arg415, Lys207, Arg56*, Tyr109, and Tyr54* have their counterparts in the CaStr2p model, namely Arg572, Lys407, Arg47, Tyr312, and Tyr45, respectively. There is little doubt that the relative spatial position of all key residues in both centres shows very significant homology, despite the fact that in CaMet15p two out of five key residues derive from the neighbouring subunit of dimeric protein, while CaStr2p is supposed to be monomeric.

**Figure 8.**
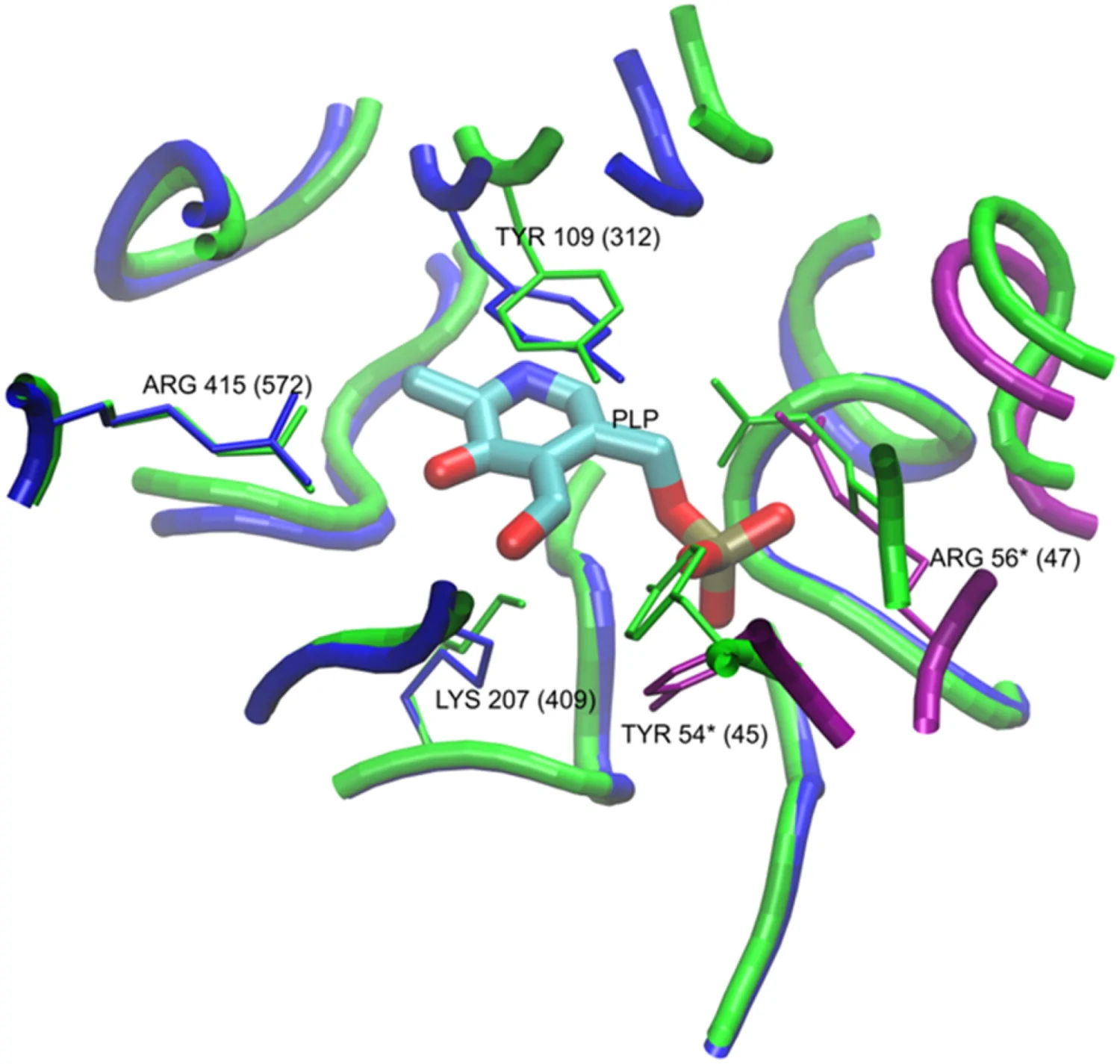
Superposed structures of CaMet15p (blue and purple) and CaStr2p (green) modelled L-OAH binding sites. Only residues within a 7 Å radius of PLP are shown as tubes. Essential residues mentioned in the text are represented as thin sticks, while the PLP cofactor is shown as thick sticks. Residues are numbered according to CaMet15p. Two residues derived from another subunit are marked with asterisk. Numbers in parentheses correspond to the equivalent residues in CaStr2p.

The strong homology of CaStr2p L-OAH binding site to that of CaMet15p suggests that mechanism of CaStr2p inhibition by BIA should be identical with that of CaMet15p. Taking into account that the initial steps of the proposed molecular mechanism of reaction catalysed by cystathionine γ-synthase^41^^,^^42^ are exactly the same as those proposed for the reaction catalysed by *O*-acetyl-L-homoserine sulfhydrylase, including the side chain oxygen atom protonation by the active site tyrosine residue (Tyr312 in CaStr2p), it seems highly probable that CaStr2p inhibition by BIA is also of the dead- end type.

The inhibitory potential of BIA towards CaStr2p was confirmed experimentally. BIA inhibited CaStr2NHp activity with IC_50_ = 1.76 mM (Supplementary materials, S5.1). The inhibition was of a competitive character in respect to L-OAH and the calculated K_i_ value was 0.912 mM (Supplementary materials, S5.2)

Homology of L-OAH binding sites in CaStr2p and CaMet15p models and efficient docking of BIA to both models convincingly explain the inhibitory properties of this compound towards real enzymes. This feature is undoubtedly advantageous for BIA as a potential antifungal agent. Simultaneous blocking of the direct sulfhydrylation and transulfuration branches of MBP ensures complete inhibition of intracellular L-Met formation, and in consequence protein biosynthesis, in BIA-treated fungal cells.

## Conclusions

The isoxazolinon-alanine derivative BIA, previously reported as a fungicide active against phytopathogenic fungi^13^^,^^14^, has now been shown to exhibit growth inhibitory activity against a number of human pathogenic yeasts of Candida spp. Studies on the mechanism of this effect in *Candida albicans* revealed that BIA is taken up by the methionine specific permease Mup1p and then inhibits two enzymes of the MBP, namely *O*-acetyl-L-homoserine sulfhydrylase Met15p and cystathionine γ-synthase Str2p, thus effectively precluding biosynthesis of L-Met. Inhibition of both Met15p and Str2p by BIA is possible, due to the structural analogy of this agent and L-OAH, a natural substrate of the two enzymes. BIA acts as a competitive inhibitor of both enzymes in respect to L-OAH, most probably of the dead-end type.

It is noteworthy that very few inhibitors of Met15p or Str2p, acting as substrate analogues, have been known so far. Kulikova et al. identified L-4-acetamido-2-aminobutanoic acid (AcDAB) and L-propargylglycine as strong inhibitors of bacterial Met15p^30^. However, in our previous studies on putative inhibitors of CaMet15p, AcDAB was found to inhibit this enzyme with IC_50_ = 4.8 mM, but appeared completely devoid of anticandidal *in vitro* activity, even in minimal media^22^. Identified inhibitors of Str2p include L-propargylglycine, targeting bacterial Str2p^43^ and 2-amino-5-phosphono-3-pentenoic acid, inhibiting the plant enzyme^44^. The former compound was reported to exhibit moderate growth inhibitory effect against plant pathogenic fungi^45^ but its possible anticandidal activity is unknown. BIA is thus the only inhibitor of both Met15p and Str2p identified so far, exhibiting moderate growth inhibitory activity against human pathogenic fungi of the Candida genus. We believe that it is possible to rationally design another dual inhibitor(s) of both enzymes with more favourable than BIA enzyme inhibitory potential and antifungal properties.

The anticandidal *in vitro* activity of BIA is good in minimal YNB media, but much lower in L-Met containing media, including the CLSI-recommended RPMI-1640 medium. The need to compete with L-Met for Mup1-mediated uptake seems to be an important limitation of BIA as an antifungal agent in this respect. Moreover, resistance to BIA may appear in clinical strains carrying mutations resulting in downregulation of *MUP1* expression or reduced affinity of Mup1p to this agent. It is worth mentioning therefore, that microbial methionine auxotrophs showed a strongly reduced ability to proliferate in human serum^46^, where the L-Met level is not higher than 50 µM^47^. Moreover, we were previously able to show that the L-Met concentration, which rescued the growth defect of *C. glabrata* cells treated with L-PEN in YNB medium was higher than 1 mM^10^. It can therefore be assumed that bypassing the Mup1 transport barrier may potentially increase the antifungal activity of BIA in methionine containing media and in human serum. One possibility to achieve such an effect could be to take advantage of the Trojan horse strategy^48^^,^^49^, *i.e.,* converting BIA into a derivative that enters the fungal cell via a pathway other than Mup1 and is then degraded in the cytosol, releasing BIA. Our further research is aimed at reaching this goal.

## Supplementary Material

BIA_JEIMC_Supplementary materials_rev.pdf

## Data Availability

The data supporting the findings of this study are available within the article and its supplementary materials. The raw spectroscopic data presented in this study are openly available in https://mostwiedzy.pl/pl/open-research-data at doi: 10.34808/vrjv-nn70
