# Supplementary material for: Anticandidal activity of L-β-(isoxazolin-5-on-2-yl)-alanine due to the inhibition of O-acetyl-L-homoserine sulfhydrylase and cystathionine γ-synthase
Source: J Enzyme Inhib Med Chem. 2026 Oct 21;41(1):2749883. doi: 10.1080/14756366.2026.2749883 (PMC13715334; doi:10.1080/14756366.2026.2749883)
Supplement: Supplementary file 1 [file IENZ_A_2749883_SM4531.pdf]

Anticandidal activity of L-β-(isoxazolin-5-on-2-yl)-alanine due to the inhibition of *O*-acetyl-L-homoserine sulfhydrylase and cystathionine γ-synthase

Supplementary materials

Content

S1. NMR spectra of BIA p.2

S2. HPLC analysis of BIA purity and HRMS spectrum p.3

S3. Electrophoretic analysis of CaStr2NHp purification p.4

S4. Determination of MW and quaternary structure of CaStr2NHp p.5

S5. Determination of IC_{50} and kinetic analysis of CaStr2NHp inhibition by BIA p.6

S1. NMR spectra of BIA

S1.1 ^{1}H NMR spectrum

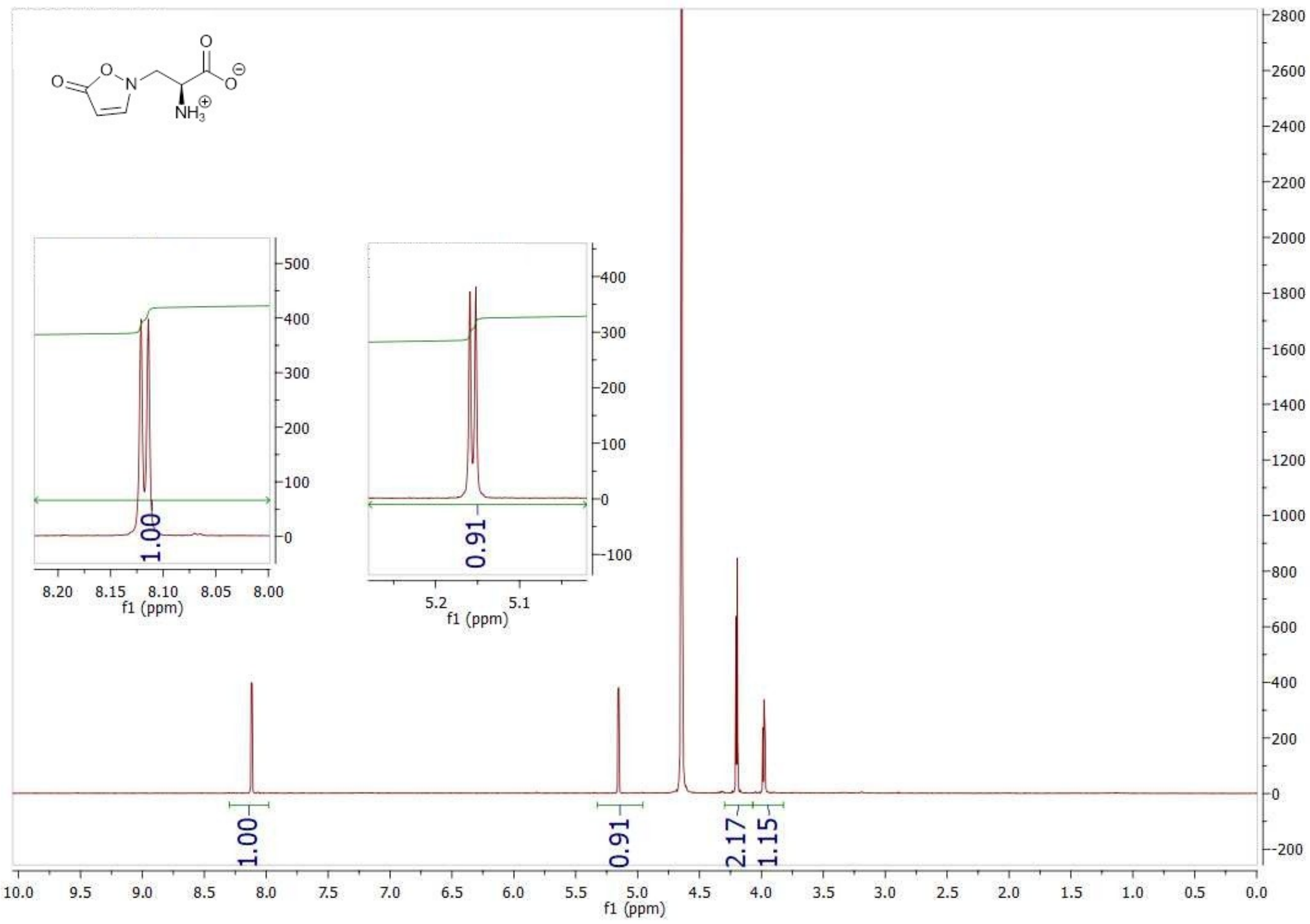

S1.2 ^{13}C NMR spectrum

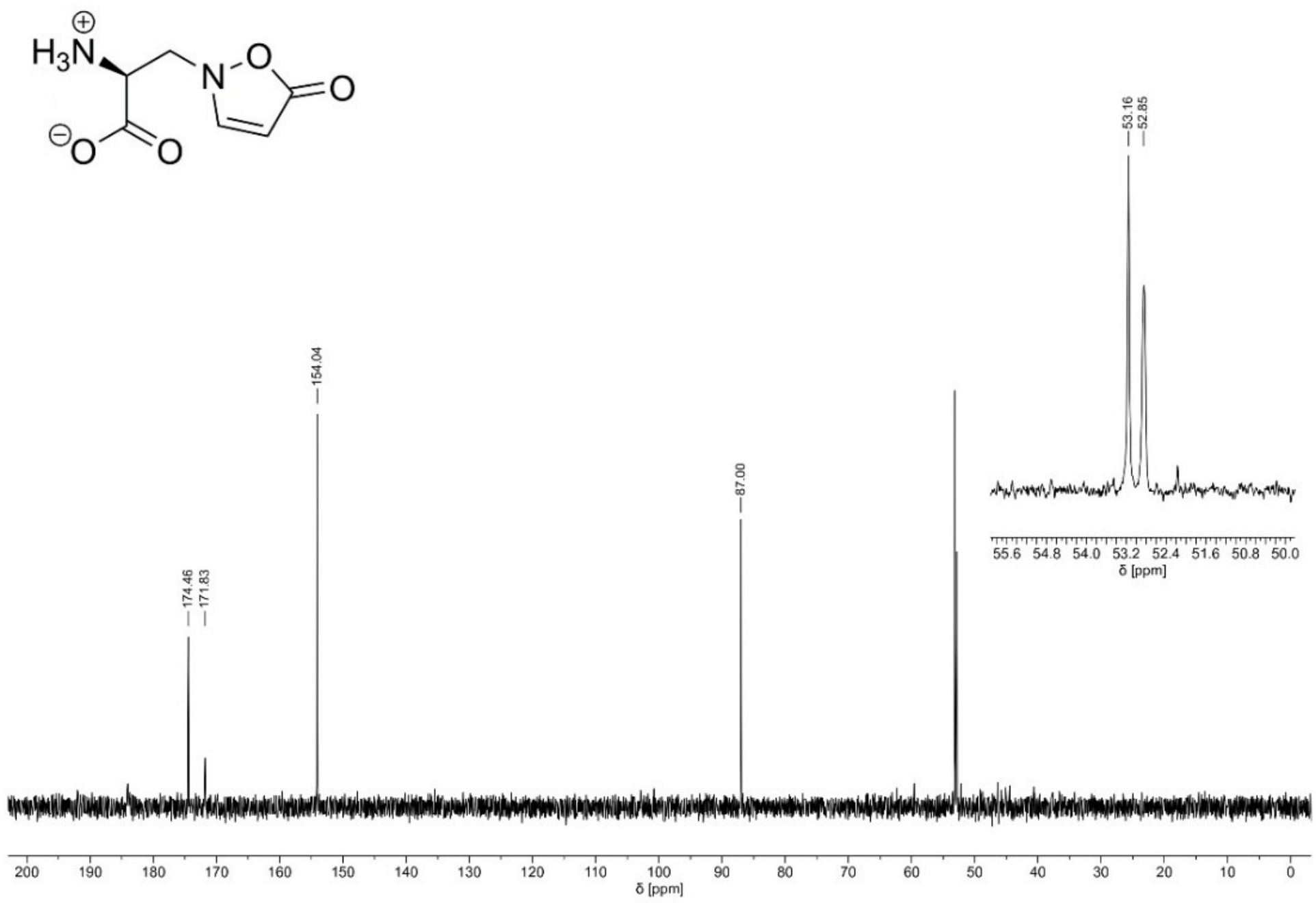

S2. HPLC analysis of BIA purity and HRMS spectrum

S2.1 HPLC analysis

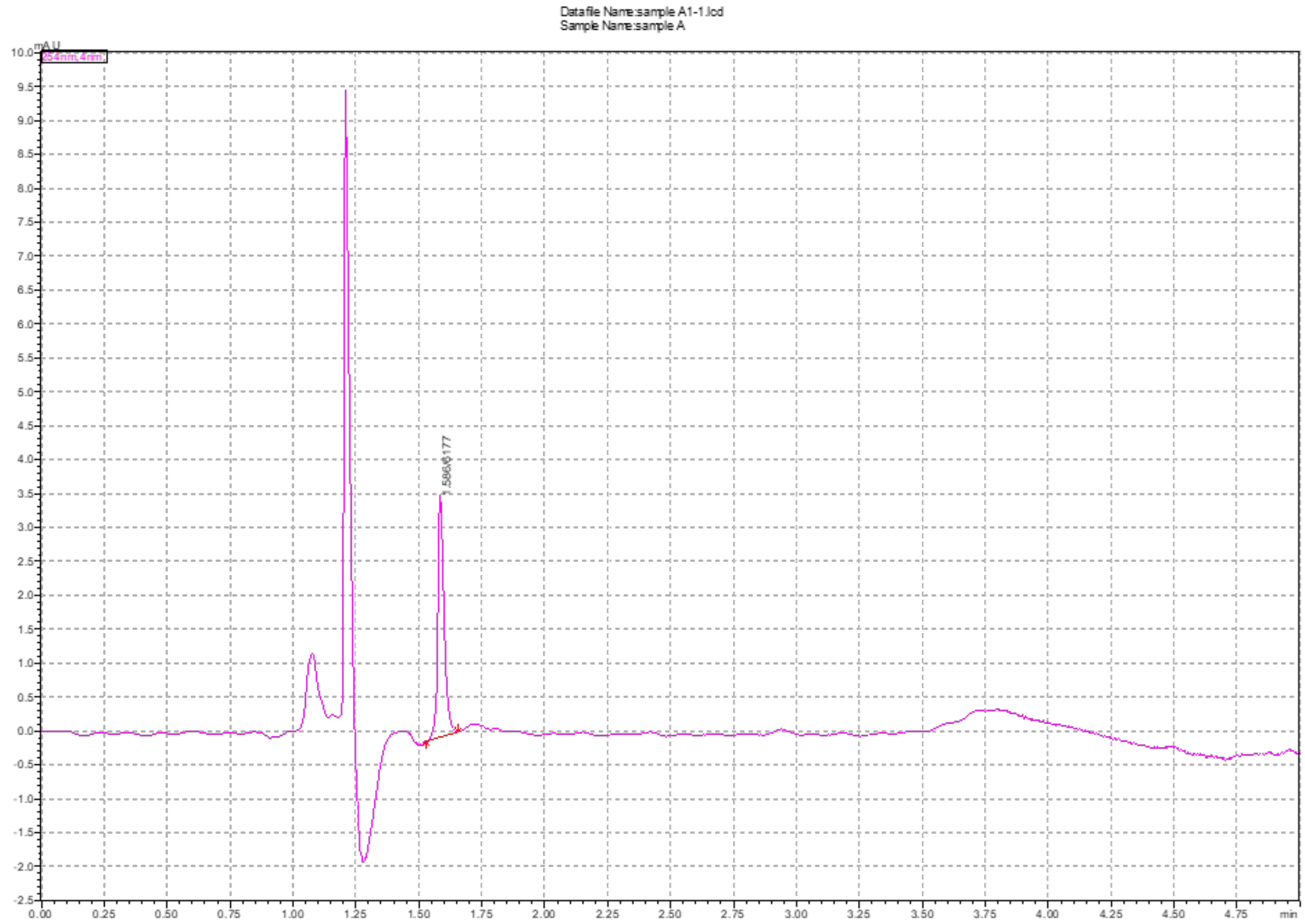

Purity: 97.4%

S2.2. HRMS spectrum

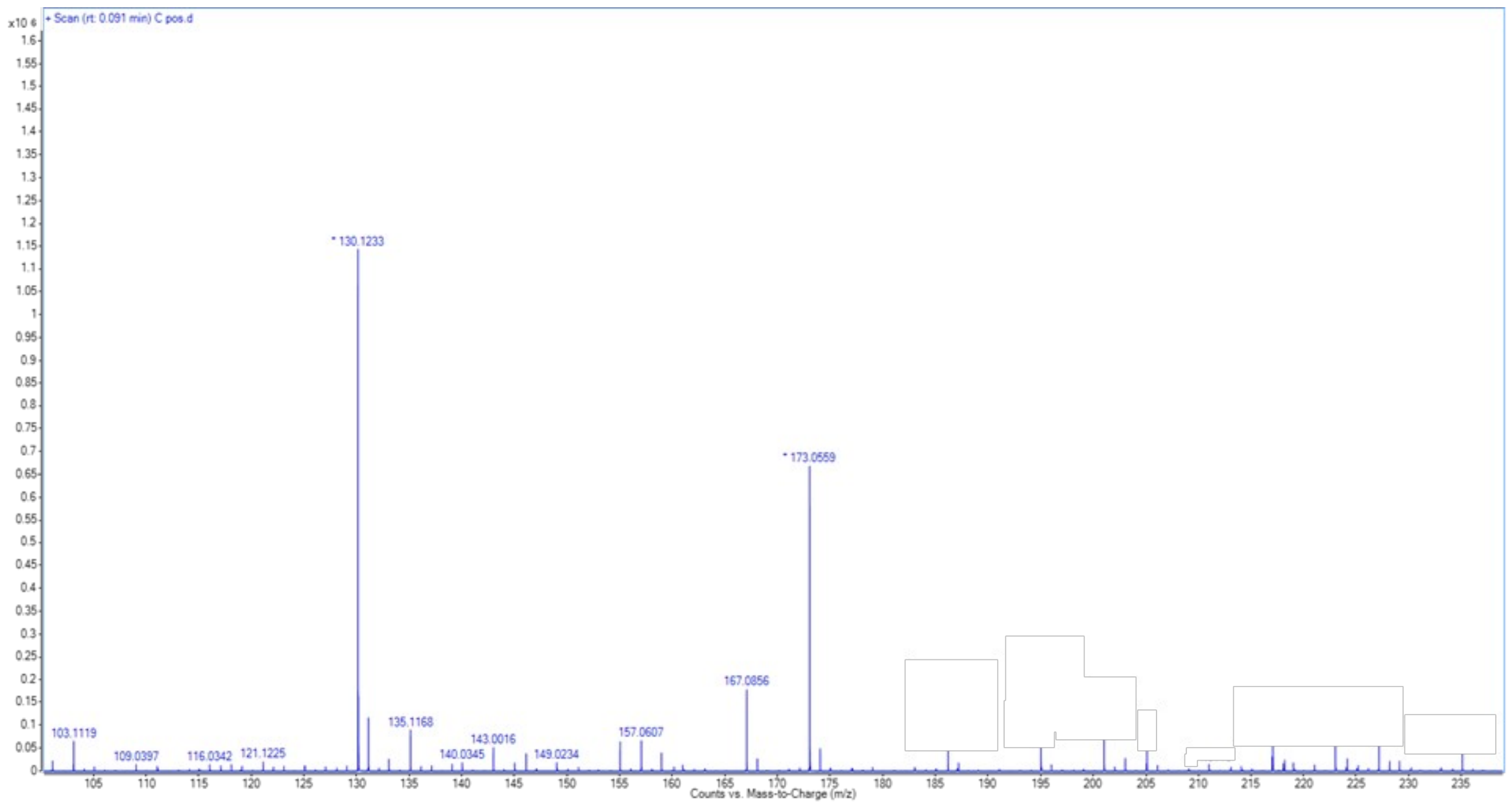

Compound stability: BIA is entirely stable in solution under physiological conditions (pH 6 – 7.5, temperature range 20 ˚C – 40 ˚C). It decomposes (ring opening) in alkaline solutions (pH>8) and in strongly acidic (pH<2) solutions.

S3. Electrophoretic analysis of CaStr2NHp purification

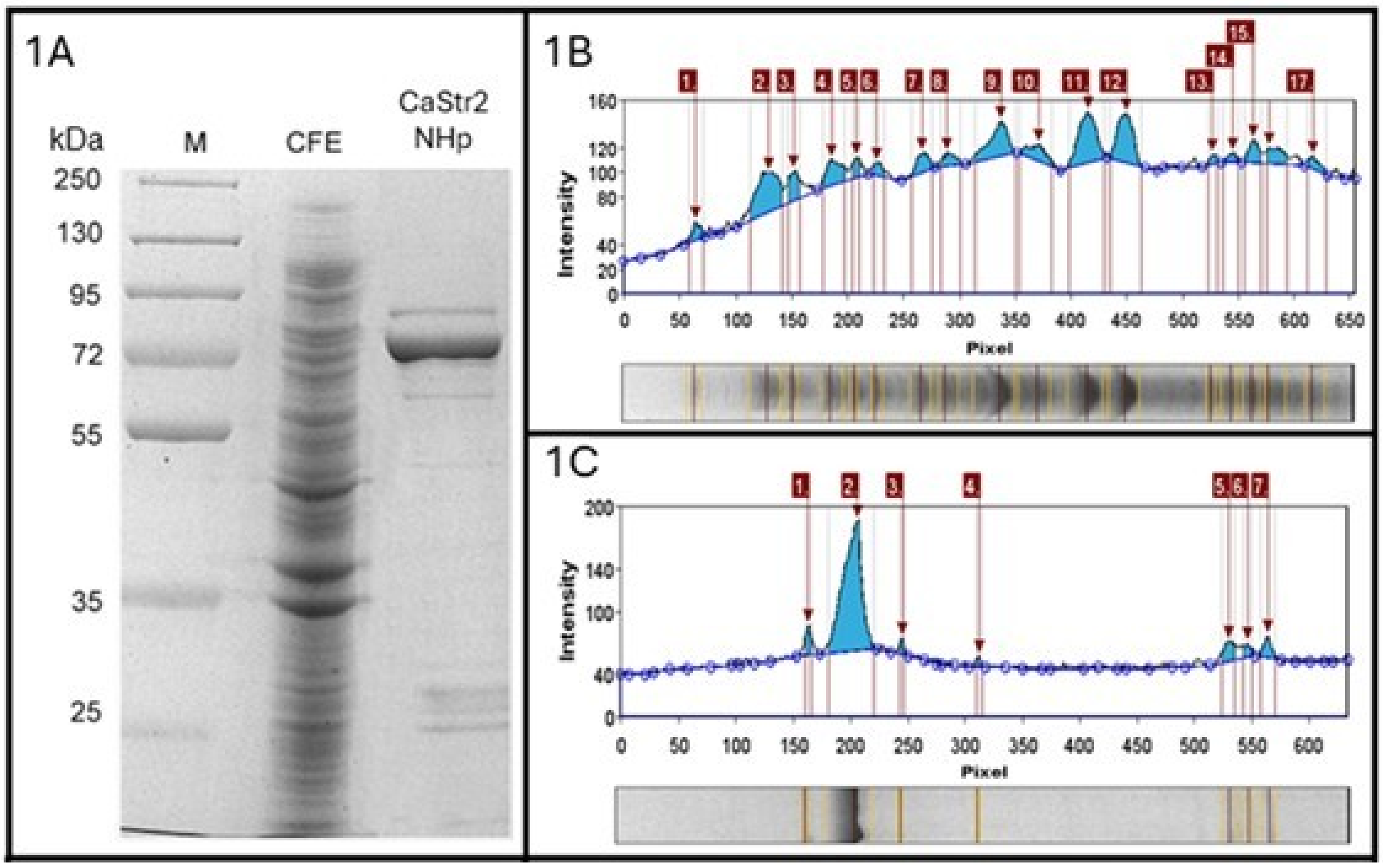

1A. SDS-PAGE analysis of cell-free extract (CFE line) from pLate52:CaSTR2NH transformed ER2566 cells and eluate from the HisTrap™ Fast Flow column (CaStr2NHp line); M line, Thermo Scientific PageRuler™ Plus Prestained Protein Ladder. 1B. Densitometric analysis of band pattern in CFE line; 1C. Densitometric analysis of band pattern in CaStr2NHp line. M, marker Thermo Scientific PageRuler™ Plus Prestained Protein Ladder (Thermo Scientific). Densitometric analysis was performed with Gel Analyzer 19.1 software.

Electrophoresis was performed at 18 V cm^{-1}, with 5% stacking gel and 10% resolving gel

S4. Determination of molecular mass and quaternary structure of CaStr2NHp

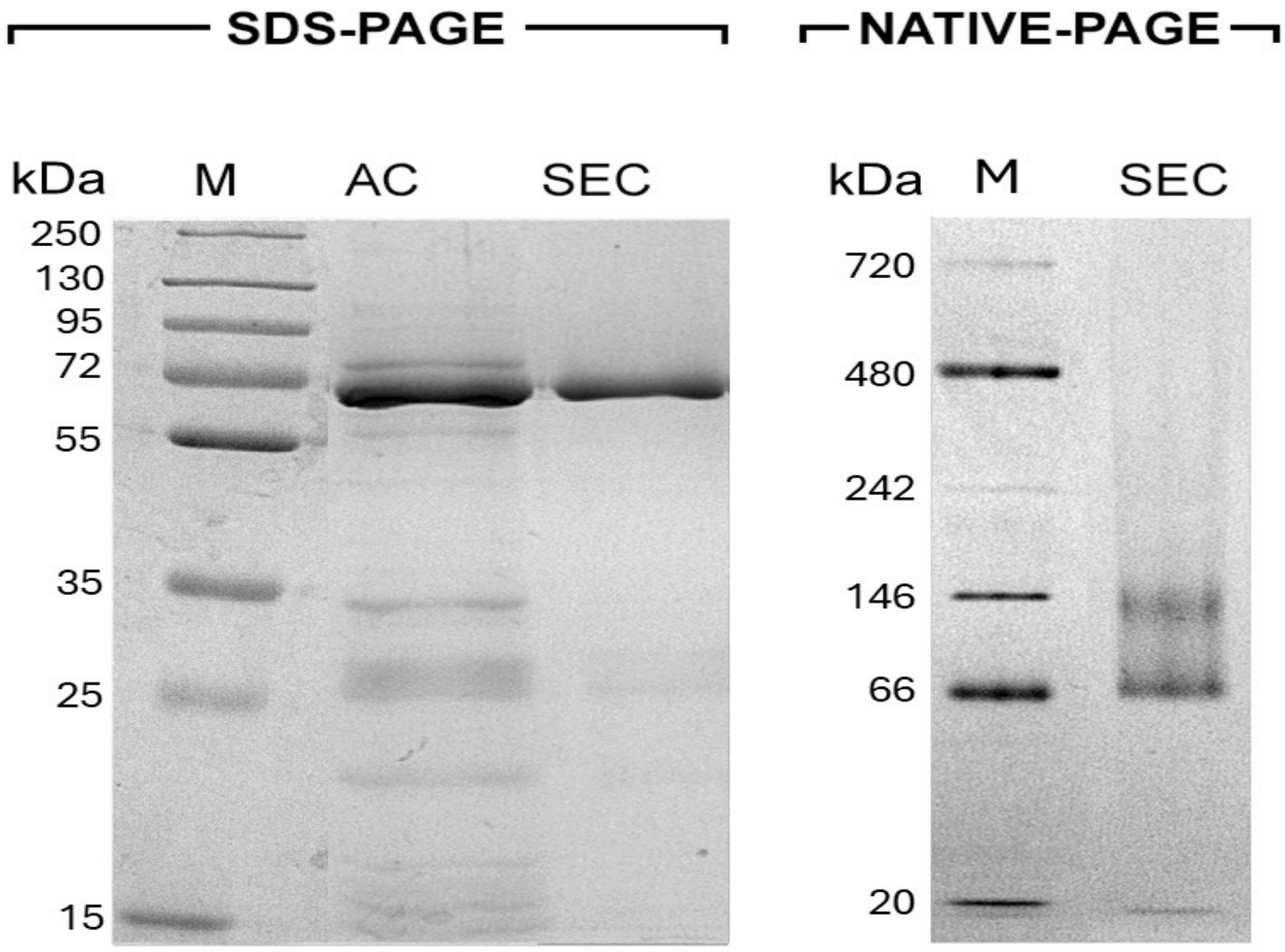

SDS-PAGE: M line, Thermo Scientific PageRuler™ Plus Prestained Protein Ladder; AC line, CaStr2NHp preparation after affinity chromatography; SEC line, CaStr2NHp preparation after size exclusion chromatography.

SDS-PAGE was performed at 18 V cm^{-1}, with 5% stacking gel and 10% resolving gel.

NATIVE-PAGE: M line, NativePAGE™ Thermo Scientific Native MARK™ Unstained Protein Standard; SEC line, CaStr2NHp preparation after size exclusion chromatography.

NATIVE-PAGE was performed with NativePAGE™ Novex Bis-Tris Gel System kit (Invitrogen), according to the given manual.

S5. Determination of IC_{50} and kinetic analysis of CaStr2NHp inhibition by BIA

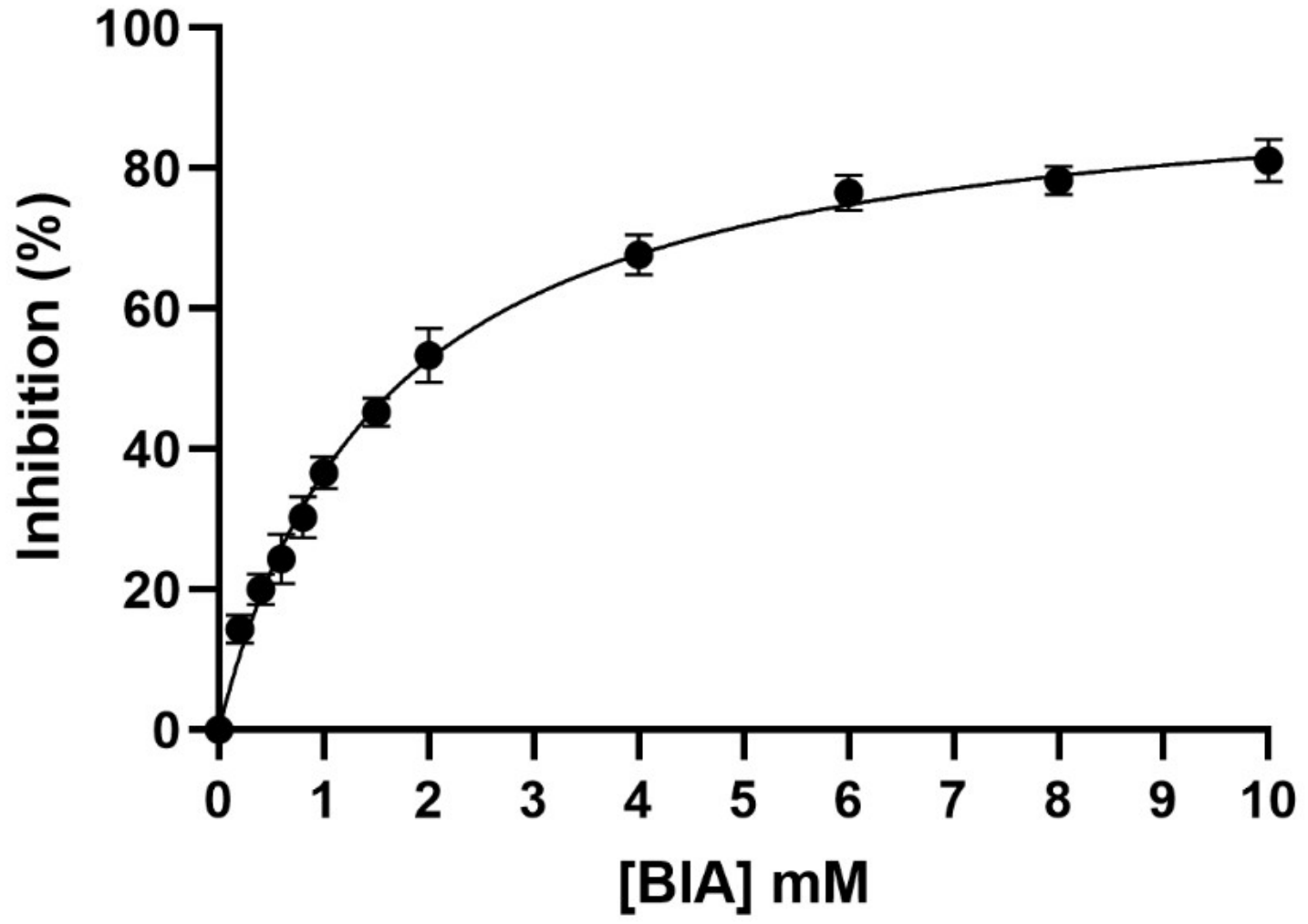

S5.1 Inhibition of CaStr2NHp activity by BIA at fixed L-OAH and L-Cys concentrations. The error bars represent ±SD.

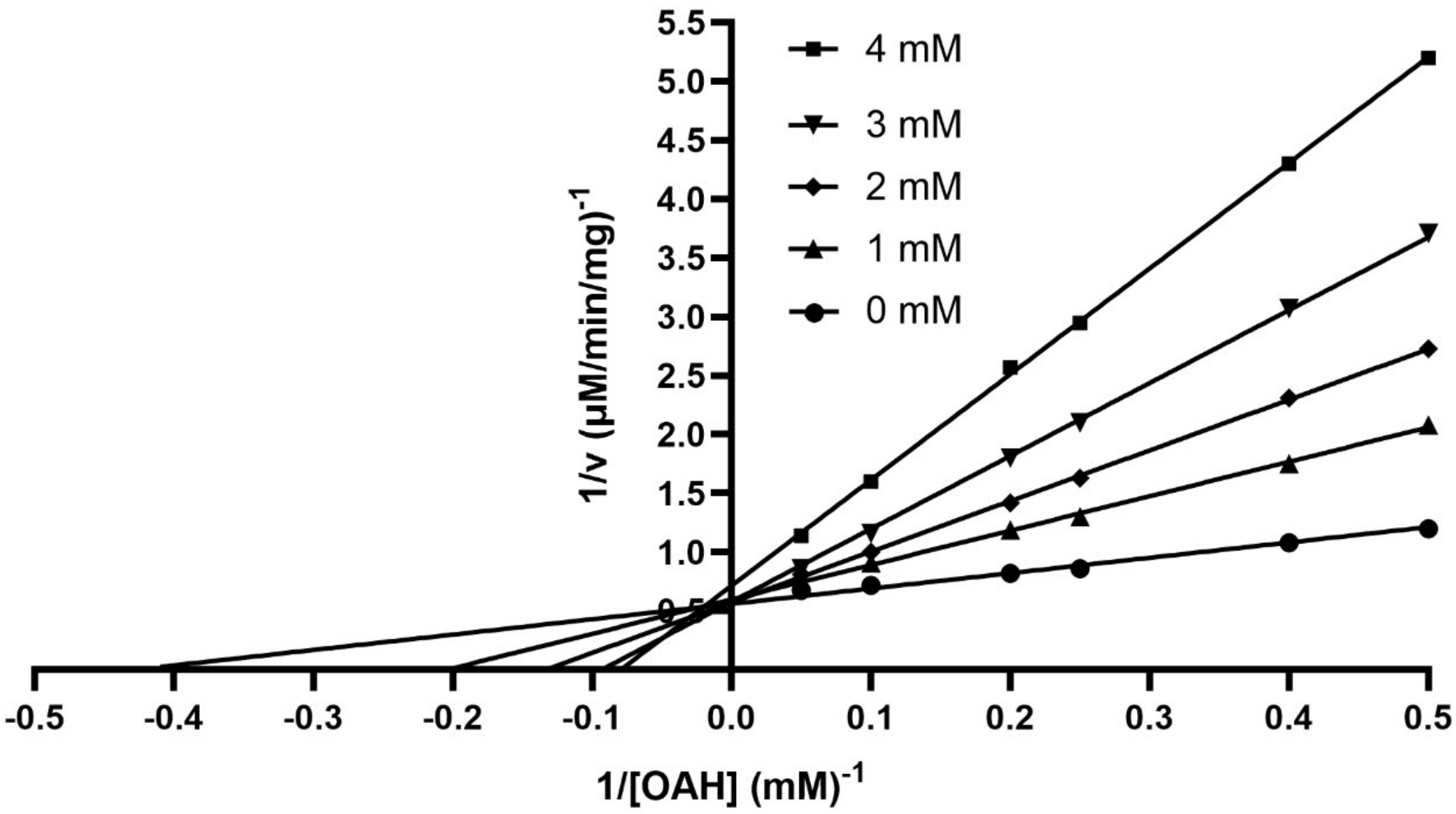

S5.2 Competitive inhibition of CaStr2NHp by BIA in respect to L-OAH. BIA concentrations are indicated inside
